# Structure and membrane interactions of *Arabidopsis thaliana* DGD2, a glycosyltransferase in the chloroplast membrane

**DOI:** 10.1016/j.jbc.2025.108431

**Published:** 2025-03-20

**Authors:** Emma Scaletti Hutchinson, Markel Martínez-Carranza, Biao Fu, Lena Mäler, Pål Stenmark

**Affiliations:** Department of Biochemistry and Biophysics, Stockholm University, Stockholm, Sweden

**Keywords:** *A. thaliana*, chloroplast, DGD2, glycolipid, glycosyltransferase, outer membrane

## Abstract

Galactolipids are characteristic lipids of the photosynthesis membranes of higher plants and cyanobacteria. Due to their close relationship to the stability of the photosystem protein complexes, the biogenesis of galactolipids has been intensively studied on the genetic and molecular levels. There are two major types of galactolipids in chloroplastic membranes: monogalactosyldiacylglycerol and digalactosyldiacylglycerol (DGDG). Under phosphate-limiting conditions, the amount of DGDG increases dramatically to allow for phosphate salvage from phospholipids. In *Arabidopsis thaliana*, the membrane-associated glycosyltransferase digalactosyldiacylglycerol synthase 2 (atDGD2) is highly responsive to phosphate starvation and is significantly upregulated during such conditions. The lipid galactosylation reactions are also fundamentally interesting as they require a catalyst that is capable of bringing a hydrophilic and lipophilic substrate together at the solution-membrane phase border. Here, we present the X-ray crystal structure of atDGD2, which is the first reported DGDG synthase structure. AtDGD2 is most structurally similar to functionally unrelated GT-B enzymes. Interestingly, in spite of significant donor substrate binding differences, we identified four amino acids (Gly22, His151, Lys243, and Glu321, atDGD2 numbering) which were entirely conserved between the structurally similar enzymes. We also investigated the membrane interaction kinetics and membrane anchoring mechanism of atDGD2. This demonstrated that atDGD2 is membrane-bound but also showed that membrane binding is highly dynamic. Furthermore, our structural information in context of previous biophysical studies highlights regions of the enzyme exhibiting a high degree of structural plasticity, which we propose to be important for allowing atDGD2 to quickly adapt its activity based on the membrane lipid environment.

Lipid galactosylation is a vital task for the homeostasis of the photosynthesis membranes. Photosynthetic membranes in chloroplasts contain around 55% monogalactosyldiacylglycerol (MGDG) and 20% digalactosyldiacylglycerol (DGDG), together with mainly phospholipids (phosphatidylcholine and phosphatidylglycerol) and sulfoquinovosyldiacylglycerol (1, 2, 3). The synthesis of galactosylated lipids (Fig. 1) is initiated by the attachment of a galactose moiety (derived from the donor substrate UDP-galactose) to diacylglycerol (DAG) through a β-glycosidic linkage; a process catalyzed by MGDG synthases (4, 5). A major percentage of MGDG is further galactosylated to DGDG, a process that is catalyzed by digalactosyldiacylglycerol synthases (4, 5). It has been shown that the addition of second galactosyl confers DGDG significantly different lipid properties compared to MGDG (6). MGDG is a nonbilayer forming lipid while DGDG is bilayer forming (6, 7), which makes the relative composition of these lipids important for the membrane homeostasis. Oligogalactolipids (TriGDG and TetraGDG) are also present in the chloroplast membranes, however only in trace amounts (8).

**Figure 1:**
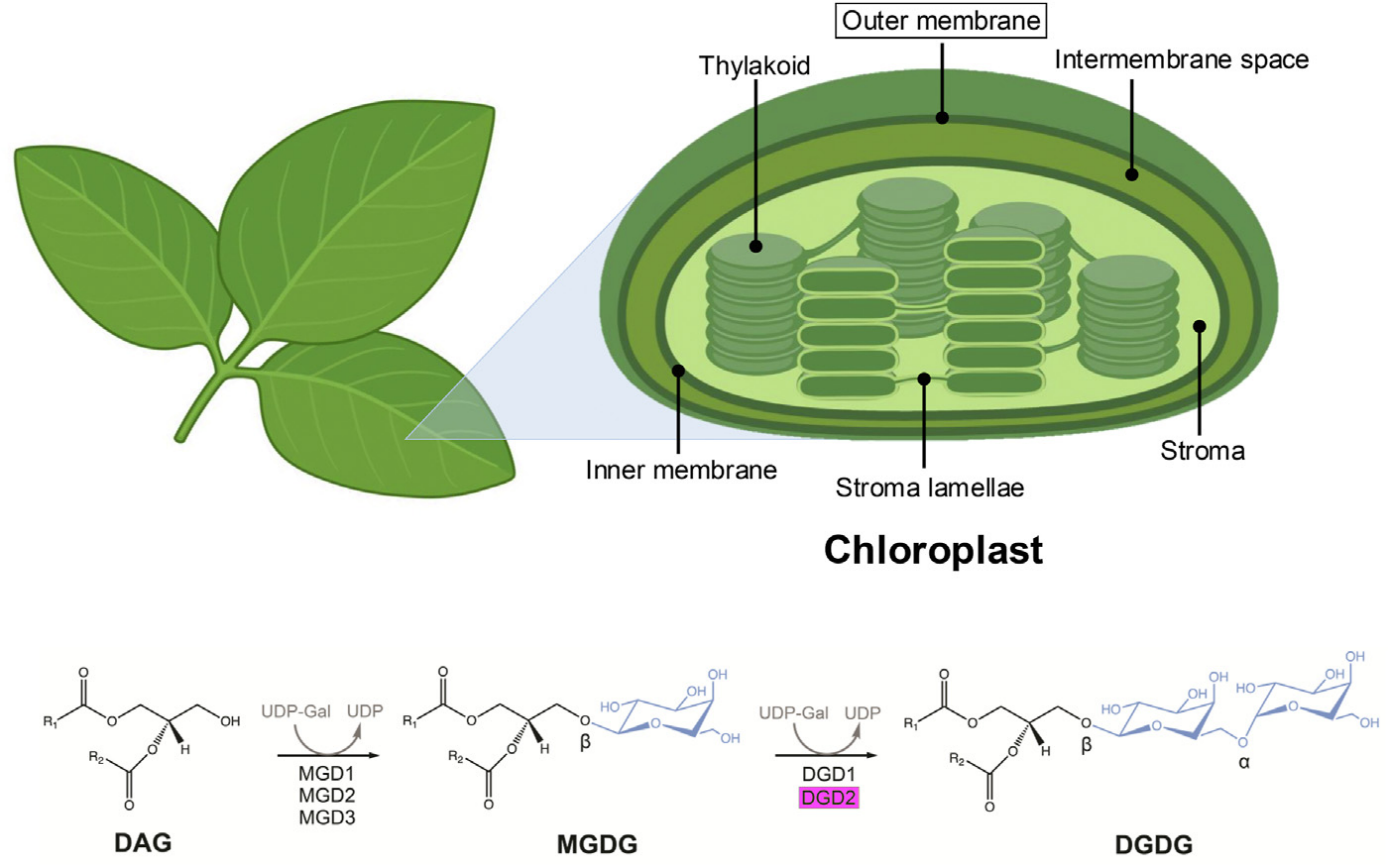
**Lipid galactosylation reactions in *Arabidopsis thaliana*.** Galactolipids such as DGDG are an important component of the outer membrane in plant chloroplast organelles. Diacylglycerol (DAG), monogalactosyldiacylglycerol (MGDG), and digalactosyldiacylglycerol (DGDG). UDP-galactose (UDP-Gal). Enzymes: monogalactosyldiacylglycerol synthase 1 (MGD1), monogalactosyldiacylglycerol synthase 2 (MGD2), monogalactosyldiacylglycerol synthase 3 (MGD3), digalactosyldiacylglycerol synthase 1 (DGD1), and digalactosyldiacylglycerol synthase 2 (DGD2). In the chemical structures, galactose residues are colored *blue*. The enzyme DGD2, the focus of this work, is highlighted by a *magenta box*. Figure produced using ChemDraw (v. 19.1) and BioRender (https://BioRender.com/u95m596).

In *Arabidopsis thaliana* (*A. thaliana*), there are two genes for DGDG synthesis, *DGD1* and *DGD2*. The overall protein sequences encoded by *DGD1* and *DGD2* are significantly different (9), that is, *DGD1* and *DGD2* code for protein sequences of 808 and 473 residues, respectively (Fig. S1*A*). However, DGD2 shares a high degree of sequence similarity with the glycosyltransferase domain of DGD1 (Fig. S1*B*). It has been indicated that *DGD1* codes for an additional domain at the N-terminal side, which is not related to enzyme function (10), but is involved in lipid transport (11). Under normal conditions, the isoform *DGD1* is responsible for producing most of the DGDG lipids. However, during phosphate shortage, *DGD2* is upregulated and synthesizes DGDG in the outer membrane of the chloroplast (9, 10, 12). In this way, DGDG can replace phospholipids that are recycled to release phosphate for more acute purposes. Therefore, DGD2 is important for the plant’s viability during stress conditions, such as nutrient shortage leading to lack of phosphate (13). For DGD2 to carry out its enzymatic function, it must become membrane integrated (9, 14). This requires that the regions in DGD2 that anchor the protein to the membrane are surface-exposed (15, 16). It has also been demonstrated that negatively charged lipids are also important for function as they increase the activity of DGD2, presumably by altering the membrane–protein interactions (15, 17).

We previously reported the expression and purification of recombinant atDGD2 in an *Escherichia coli*–based system (17). In order to obtain pure, homogenous and highly active atDGD2 it was necessary to remove around 70 amino acids from the C terminus of the protein. Importantly, it was shown that the deleted C-terminal residues were not required for atDGD2 activity. This deleted sequence is likely required for DGD2 targeting/sorting to the chloroplast and is cleaved off to produce mature atDGD2 protein *in vivo* (17). AtDGD2 belongs to the GT4 family of glycosyltransferase (GT) enzymes and is predicted to have at GT-B fold, where two Rossmann-like domains are connected via a flexible linker (14). Currently the only existing atDGD2 structural information pertains to a single alpha-helix (residues 227–245), which was solved by Solution NMR (Protein Data Bank [PDB] ID: 2L7C) (16). A sequence search of atDGD2 in the PDB did not identify any proteins (other than the aforementioned atDGD2 alpha-helix) with a similar amino acid sequence that have had their structures determined. However, while no structures of DGD2 are available, a structure of atMGD1 which catalyzes the first step of DGDG synthesis (Fig. 1) has been solved (18). Determining the structure of atDGD2 would therefore provide important insights and a more complete picture of the DAG lipid galactosylation process in *A. thaliana*.

Herein, we present the X-ray crystal structure of the integral monotropic membrane protein atDGD2, bound to its donor nucleotide-sugar substrate UDP-galactose. AtDGD2 was shown to be most structurally similar to functionally unrelated proteins from the GT-B fold superfamily. Detailed analysis revealed important conserved residues between these structures and also revealed structurally dynamic regions in atDGD2 that are likely responsible for membrane interactions. Such structural plasticity may be an important means by which atDGD2 can rapidly adapt its activity based on the lipid bilayer environment. Moreover, *in vitro* membrane binding kinetic studies showed that the enzyme is almost completely membrane associated and also revealed that the membrane association is highly dynamic. Overall, the data presented here provide insights in the fundamental processes of lipid galactosylation in *A. thaliana.*

## Results

### Overall structure of atDGD2

**Figure 2:**
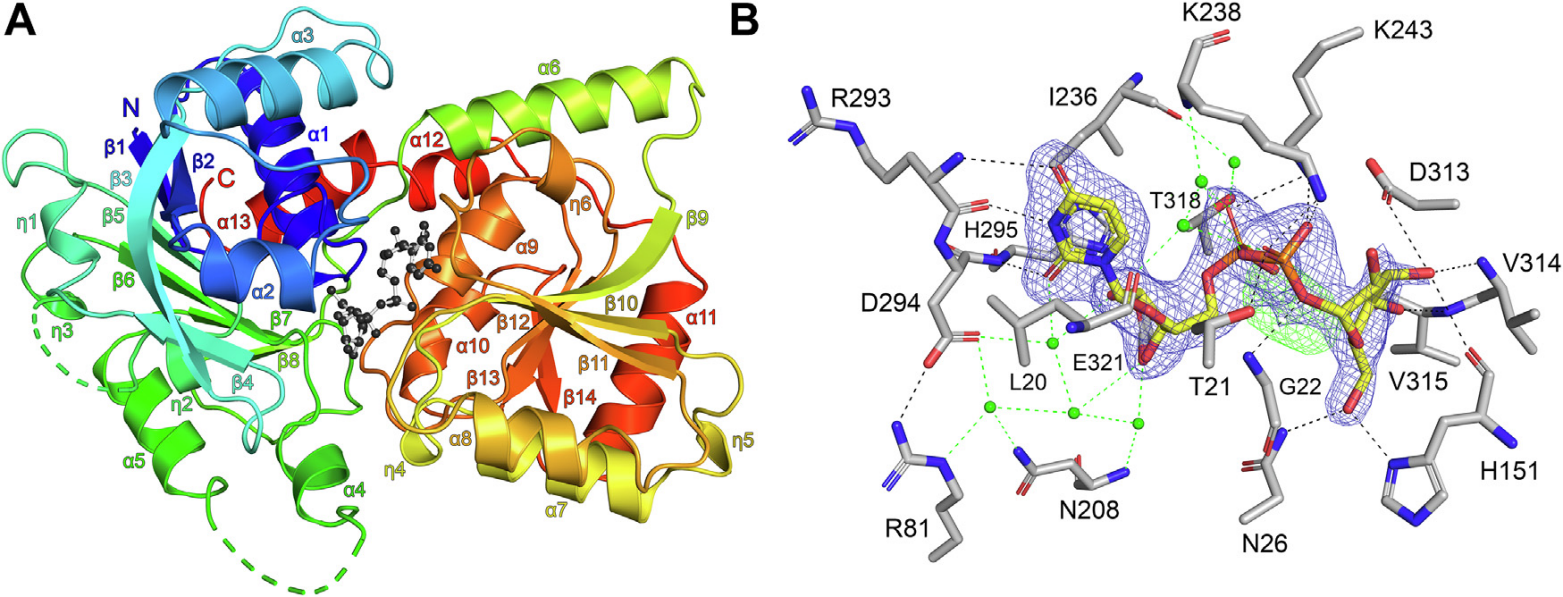
**Crystal structure of atDGD2 in complex with UDP-galactose.***A*, atDGD2 monomer shown as a *rainbow cartoon* with secondary structure annotation. UDP-galactose is shown as a *ball-and-stick model*. *B*, hydrogen bond network for UDP-galactose in the atDGD2 active site. Amino acids involved in ligand coordination are depicted as *sticks*. Hydrogen bond interactions are shown as *dashed lines*. Water mediated hydrogen bonds are shown as *green dashed lines*. The 2*F*_o_−*F*_c_ electron density map around UDP-galactose is contoured at 1.0 *σ* (*blue*), and the *F*_o_−*F*_c_ electron density maps are contoured at +3.0 *σ* (*green*) and −3.0 *σ* (*red*). UDP-galactose is depicted as a *stick model*; C atoms are colored *yellow*, O atoms *red*, N atoms *blue*, and P atoms *orange*. Figures were produced with PyMOL (v.2.3.3, Schrödinger) (https://pymol.org).

The X-ray crystal structure of atDGD2 in complex with its substrate UDP-galactose was solved in space group *P*4_1_2_1_2 to 2.10 Å resolution. The protein crystallizes as a monomer in the asymmetric unit. This is consistent with previous reports which have shown that atDGD2 is also monomeric in solution (17). Data collection and refinement statistics are shown in Table S1. The atDGD2 monomer has a classic GT-B fold (19, 20, 21, 22), comprised of two distinct Rossmann-like β/α/β domains connected by a flexible loop region. The N-terminal domain (residues 1–201 and 380–391) consists of a six-stranded twisted β-sheet (β1-2 and β5-8), which is surrounded by two β-strands (β3-4), three 3_10_-helixes (η1-3), five α-helices (α1-5 and α15), as well as an additional α-helix (α15) which crosses over from the C-terminus to interact with the N-terminal domain. The C-terminal domain (residues 204–379) is comprised of a six-stranded twisted β-sheet (β9-14), flanked on both sides by three 3_10_-helix (η4-6) and seven α-helices (α6-12) (Fig. 2*A*). In the N-terminal domain there are two areas of missing electron density corresponding to residues 133 to 137 and 163 to 169. Comparison of AtDGD2 with a structural model generated using the AlphaFold webserver (23), shows that missing region 133 to 137 is an unstructured loop in the AlphaFold model, whereas missing regions 163 to 169 is largely α-helical, which significantly increases the length of helix α5 (Fig. S2). However, in the absence of lipids, this missing area is likely not helical, as is observed in our AtDGD2 structure.

### Donor sugar binding site (UDP-galactose recognition by atDGD2)

The atDGD2 structure showed clear electron density for UDP-galactose, which binds the enzyme in the anti glycosidic conformation (Fig. 2*B*). The nucleotide sugar binds in a deep cleft between the individual N- and C-terminal domains (Fig. 2*A*). Analysis of the electrostatic potential of the substrate binding cavity of atDGD2 shows that the area of the pocket, where UDP-galactose binds is predominantly positively charged, whereas the rest of the pocket is electroneutral, with small positively and negatively charged spots closer to the protein surface (Fig. S3). The ligand is positioned by residues from both the N- and C-terminal domains of the protein. The uracil base is supported by hydrogen bonding between the O4 atom and the backbone nitrogen of Arg293, the O2 atom and mainchain atoms of both Arg293 and His295, and the N3 atom and the mainchain oxygen of Arg293, in addition to hydrophobic interactions with Leu20 and Ile236. The ribose moiety is positioned by hydrogen bonding between the O2D and O3D atoms with the sidechain oxygens of Glu321, as well as a water-mediated hydrogen bond with Asp294. The diphosphate group is supported by hydrogen bonding between the α-phosphate O2A atom with the sidechain of Lys243, and the β-phosphate atoms OA2 with the sidechain of Lys243, O2B with the sidechain atoms of Thr21, and Lys238 and O3B with the mainchain nitrogen of Gly22. Lastly, the galactose of the nucleotide sugar is positioned by hydrogen bonds between its atoms O2′ with the sidechain of Lys238, O3′ with the sidechain of Asp313 and the mainchain atoms of His151 and Val314, O4′ with the mainchain atoms of Val315, and O6′ with the sidechains of Asn26 and His151. Furthermore, UDP-galactose is additionally supported by extensive water mediated hydrogen bonds with Leu20, Arg81, Asn208, Ile236, and Thr318 (Fig. 2*B*). Following the refinement of UDP-galactose in the atDGD2 active site, the *F*o-*F*c electron density map indicated a strong positive peak close to the α-phosphate of the nucleotide sugar (Figs. 2*B*, S4*A*). An additional refinement where UDP was modeled into the binding pocket together with UDP-galactose nicely resolved this additional electron density (Fig. S4*B*). This may indicate partial hydrolysis of the nucleotide donor sugar during cocrystallization.

### AtDGD2 chloroplast membrane association model

**Figure 3:**
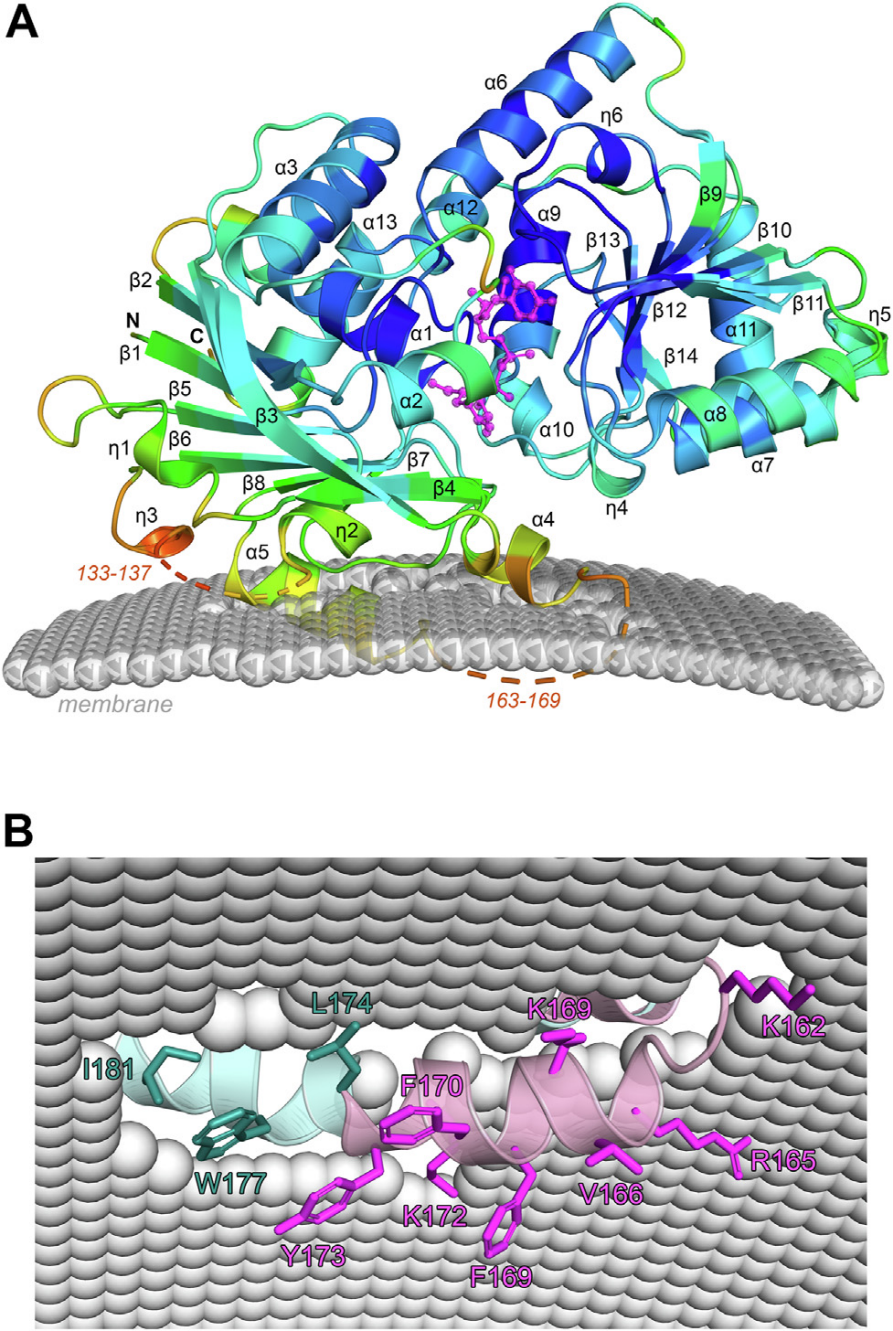
**Interaction of atDGD2-UDP-glactose with the chloroplast outer membrane.***A*, membrane interaction model created using the Orientation of proteins in membranes server (https://opm.phar.umich.edu/ppm_server3_cgopm/). The atDGD2 monomer is shown as a *cartoon* with secondary structure annotation, colored according to *B*-factor. UDP-galactose is shown as a *magenta ball-and-stick model* for clarity. In the structure the *B*-factors range from 27.7 A^2^ to 118.4 A^2^, with an average value of 57.3 A^2^ for the whole protein. The *B*-factors are depicted on the structure in *dark blue* (lowest *B*-factor) through to *red* (highest *B*-factor). AtDGD2 is predicted to interact with the chloroplast outer membrane through its N-terminal domain. Two segments of missing density (residues 133–137 and 163–169) from the atDGD2 structure are indicated. *B*, view from the underside of the membrane, highlighting important amino acids from the membrane interacting helix α5. As there is not complete electron density for this helix in the atDGD2 X-ray crystal structure (shown in *cyan*), this helix is merged with α5 from an AlphaFold3 model of atDGD2 (shown in *pink*). Figures were produced with PyMOL (v.2.3.3, Schrödinger).

In order to gain insights into how atDGD2 interacts with the chloroplast outer membrane, we generated a model using the computational tool Orientation of proteins in membranes server (http://opm.phar.umich.edu/ppm_server3_cgopm/). This suggests that atDGD2 interacts with the outer membrane via its N-terminal domain, primarily through α-helix 5 (Fig. 3*A*). This was also observed when the same analysis was performed using the atDGD2 AlphaFold model (Fig. S5). However, in our atDGD2 crystal structure, α-helix 5 is buried deeper into the membrane compared to the AlphaFold model. As noted earlier, atDGD2-UDP-galactose is missing electron density for amino acids 163 to 169, which AlphaFold predicts forms part of this important membrane interacting α-helix. *B*-factor analysis shows that the areas around the missing density in atDGD2 have the highest temperature factors in the structure (Fig. 3*A*), which suggests that the absence of electron density is a result of structural flexibility. Detailed analysis of α-helix 5 from the underside of the chloroplast membrane indicates an abundance of hydrophobic residues (Tyr166, Phe169, Phe170, Tyr173, Leu174, Trp177, and Ile181) as well positively charged amino acids (Lys162, Arg165, Lys169, and Lys172) (Fig. 3*B*), which may promote the interaction of atDGD2 with negatively charged anionic lipids in the bilayer.

### Comparison of atDGD2 with structurally related GTs

**Figure 4:**
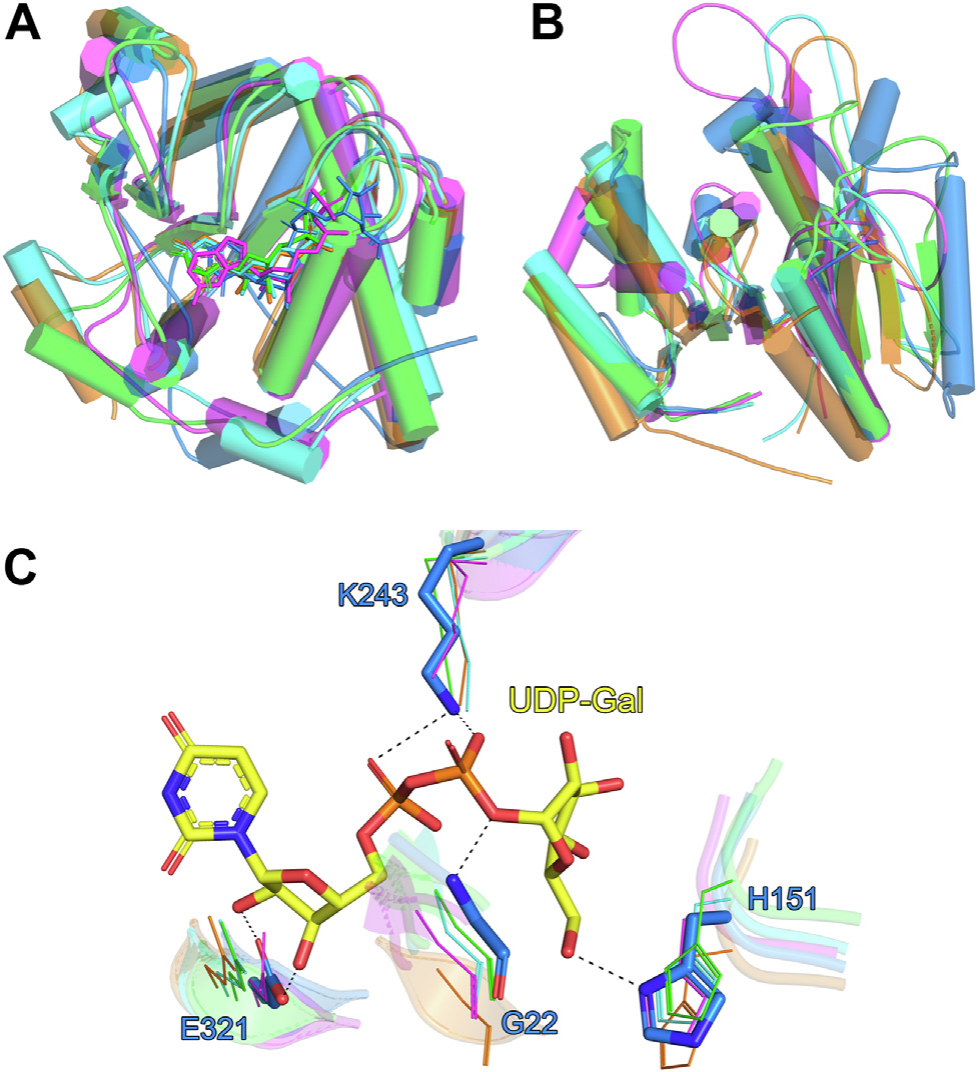
**Comparisons of the atDGD2 with structurally related glycosyltransferases.** Cα atom superpositions of the atDGD2 (*blue*) (*A*) C-terminal domain and (*B*) N-terminal domain with those from *Thermosynechococcus elongatus* SPS (*green*), *Mycobacterium smegmatis* PimA (*magenta*), *Bacillus subtilis* BshA (*orange*), and *Staphylococcus aureus* glycosyltransferase BshA (*cyan*). In *panel A* the nucleotide sugar donor substrates of each enzyme are shown as *sticks*. In the *cartoon representation*, α-helices are shown as *cylinders*. The residue ranges used for the superpositions are listed in Table S3. (*C*) Conserved amino acids in the GT structures, highlighting four important residues (atDGD2 numbering). Amino acids from atDGD2 are depicted as *blue sticks*, whereas those from the other GT structures are shown as *lines* (coloring the same as in *panels A and B*). Hydrogen bond interactions are shown as *dashed lines*. UDP-galactose from atDGD2 is depicted as a *stick model*; C atoms are colored *yellow*, O atoms *red*, N atoms *blue*, and P atoms *orange*. For clarity, the nucleotide ligands from the other structures are not shown. Figures were produced with PyMOL (v.2.3.3, Schrödinger). GT, glycosyltransferase; SPS, sucrose-phosphate synthase.

A structural similarity search was performed using the DALI webserver (24), which indicated that atDGD2 is most structurally similar to GTs with a GT-B fold, from the GT-4 family. Specifically, atDGD2 showed the highest Z-scores with *Bacillus subtilis* GT BshA (bsBshA) (25), *Staphylococcus aureus* BshA (saBshA) (26), *Thermosynechococcus elongatus* sucrose-phosphate synthase (teSPS) (27), and *Mycobacterium smegmatis* phosphatidyl mannosyltransferase (PimA) (28). Cα-atom superpositions of atDGD2 with these GTs indicated that whole monomers did not superimpose as well as the individual N- and C-terminal domains of the proteins, as indicated by their significantly higher RMSD values (Tables S2 and S3). This is in part due to differences in the relative orientation of the domains in these structures. Overall, the core structures of the individual N- and C-terminal domains are quite similar for these GTs, with the C-terminal domains superimposing somewhat better, as indicated by the slightly lower RMSD values for their corresponding Cα-atoms (Table S2, Fig. 4*A*). This partly results from significant differences in loop regions in the structures, which are more prevalent in the N-terminal domain leading to larger variability in the structures (Fig. 4*B*).

In spite of the strong structural similarity that atDGD2 shares with bsBshA, saBshA, PimA, and teSPS, the enzymes have a very low degree of amino acid sequence identity (between 11 and 13%). Furthermore, these enzymes use a diverse range of donor and acceptor substrates (Table S2), a feature common to members of the GT-B fold superfamily (14). The nucleotide diphosphate sugar substrates of each protein are primarily supported by residues from their C-terminal domains and are located within the same general area of the donor substrate binding pocket (Fig. 4*A*). All of the enzymes utilize a UDP sugar as the donor substrate, with the exception of PimA which uses a GDP sugar (Table S2). Detailed analysis of the nucleotide binding pocket of each GT indicated that the amino acids involved in substrate donor recognition are not well conserved. However, there were four residues (Gly22, His151, Lys243, and Glu321, atDGD2 numbering) which were shown to be entirely conserved between the structures (Figs. S6 and S7). These residues are important for positioning either the ribose (Glu321), phosphate group (Gly22 and Lys243), or galactose (His151) moieties of atDGD2 (Fig. 4*C*, Table S4).

### Structural comparison of atDGD2 with atMGD1

**Figure 5:**
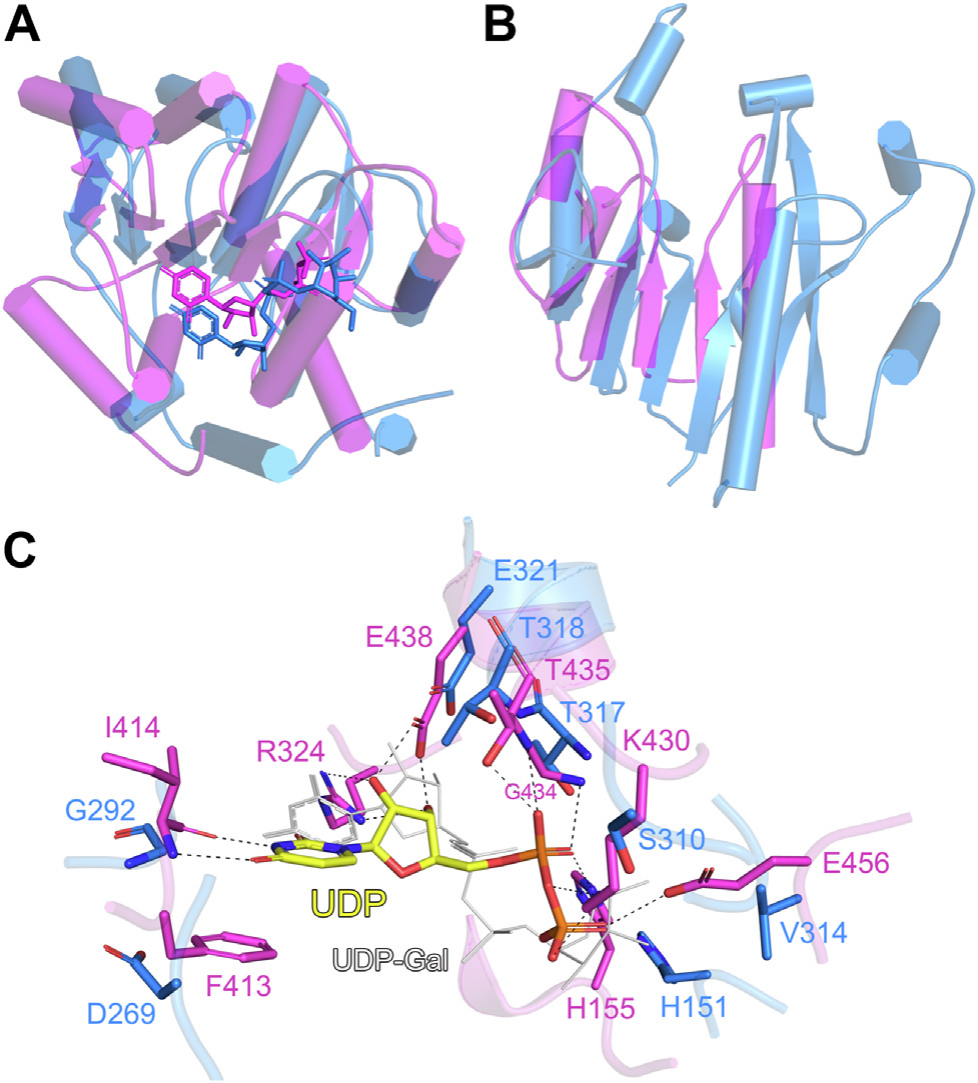
**Comparison of the atDGD2 with atMGD1.** Cα atom superpositions of the atDGD2 (*blue*) (*A*) C-terminal domain and (*B*) N-terminal domain of atMGD1 (*magenta*, PDB ID: 4X1T). In *panel A* the nucleotide donor substrates of each enzyme are shown as *sticks*. In the *cartoon representation*, α-helices are shown as *cylinders*. The residue ranges used for the superpositions are listed in Table S3. (*C*) Hydrogen bond network for UDP in the atMGD1 active site. Amino acids involved in ligand coordination are depicted as *sticks*. Hydrogen bond interactions are shown as dashed lines. UDP from the atMGD1 structure is depicted as a *stick model*; *C* atoms are colored *yellow*, O atoms *red*, N atoms blue, and P atoms *orange*. UDP-galactose from the atDGD2 structure is shown as a *thin gray stick model*. Figures were produced with PyMOL (v.2.3.3, Schrödinger).

Prior to this research, there was no structural information for atDGD2 available. However, the structure of atMGD1, the enzyme which catalyzes first step of lipid galactosylation in *A. thaliana*, had previously been solved (18). Like atDGD2, atMGD1 also requires UDP-galactose as a sugar donor, which it uses to convert DAG to MGDG, the latter of which is further processed by atDGD2 to produce DGDG (Fig. 1). The structure of atMGD1 has been solved in the presence (PDB ID: 4WYI) and absence (PDB ID: 4X1T) of UDP. Comparison of those structures showed that no major conformational changes occur upon UDP binding (18). The results for the DALI structural similarity search (24) performed for atDGD2 indicated that atMGD1 is much less structurally similar to atDGD2 compared to teSPS, bsBshA, saBshA, and PimA. Significantly higher RMSD values were observed when the Cα-atoms of either whole monomers or the individual N- and C-terminal domains in atDGD2 were superimposed (Table S2) with atMGD1 compared to the other enzymes. As was the case for the comparisons with the other GT enzymes, atMGD1 has low amino acid sequence identity with atDGD2 (11%). Furthermore, the individual domains of atMGD1 superimposed with those of atDGD2 significantly better than whole monomers, with the C-terminal domains superimposing best (Table S2). Analysis of the C-terminal domains indicate large differences in regions where UDP binds in the structures, with significant shifts in several loop structures around the ligand (Fig. 5*A*). Comparison of the N-terminal domains indicates that a large part of the atMGD1 N-terminal domain (residues 180–244) is missing in the structure (Fig. 5*B*). It should be noted that the construct of atMGD1 used to determine the structure (residues 139–526) is also additionally truncated at the N-terminal end, and the overall native enzyme is 132 amino acids longer than atDGD2. Detailed analysis of UDP binding in atMGD1 shows that the hydrogen bonding network for the ligand is very different compared to atDGD2 and the other GTs. A particularly interesting feature of the atMGD1 active site is a phenylalanine residue (Phe413) which forms a π-stacking interaction with the uracil base of UDP, a feature not present in the other structures (Figs. S6 and S7). However, the glutamate in atDGD2 which hydrogen bonds with the ribose of UDP (Glu321) is conserved in atMGD1, where the residue Glu438 performs the same function (Fig. 5*C*).

### Galactolipid acceptor substrate binding site

**Figure 6:**
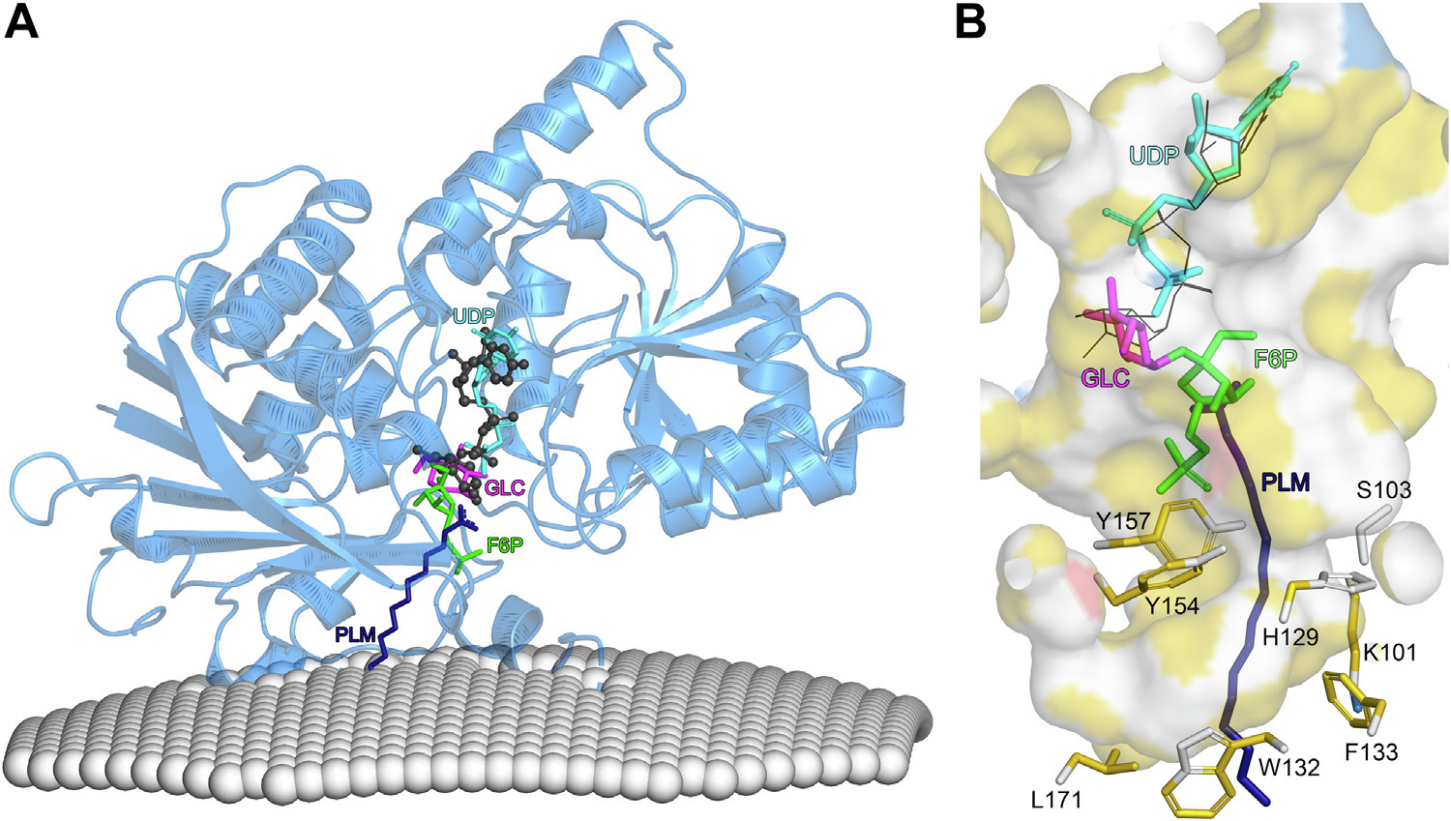
**Model of atDGD2 lipid acceptor binding.** (*A*) A model approximating MGDG galactolipid binding was produced with the AlphaFold webserver (https://alphafold.ebi.ac.uk) using the amino acid sequence of atDGD2-Δ401-473 (UniProt: Q8W1S1) as the input and selecting the ligand palmitic acid (PLM), which was most chemically similar to MGDG from the list of the available ligands. The resulting model was superimposed with the atDGD2-UDP-galactose membrane interaction model shown in Figure 3*A*. AtDGD2-UDP-galactose (shown as a *blue cartoon*) was then superimposed with glycosyltransferase teSPS (PDB ID: 6kih); only the ligands UDP (*cyan*), glucose (GLC, *magenta*), and fructose-6-phosphate (F6P, *green*) from the teSPS active site are shown in *panel A* for clarity. The teSPS reaction product S6P is a combination of GLC and F6P. The donor sugar substrate UDP-galactose from atDGD2 is shown as a *dark gray ball and stick model*. The fatty acid PLM from the AlphaFold 3 model is shown as a *dark blue stick model*. (*B*) Ligand binding cavity of atDGD2, colored according to the YRB highlighting scheme (59). Hydrocarbon groups without polar substitutions are colored *yellow*, positively charged functional groups of lysine and arginine are colored *blue*, negatively charged oxygens of glutamate and aspartate are colored *red*, while the remaining atoms, including the polar backbone, are colored *white*. The sidechains of residues from atDGD2 in close proximity to PLM are shown. In this panel, UDP-galactose from atDGD2 is shown as *thin black lines*. Figures were produced with PyMOL (v.2.3.3, Schrödinger). teSPS, *Thermosynechococcus elongatus* sucrose-phosphate synthase.

In order to gain insights into acceptor substrate binding in atDGD2, we used the AlphaFold 3 webserver (23) to generate a model of the protein bound with palmitic acid (PLM), which contains a fatty acid chain similar in that of the enzyme’s natural substrate MGDG (Fig. 6*A*). This model was then superimposed with our atDGD2 membrane interaction model (Fig. 3*A*), which if accurate, implies that the galactolipid substrate is pulled out of the membrane toward the enzyme’s donor sugar substrate UDP-galactose (Fig. 6*A*). The long hydrocarbon chain of PLM is nicely accommodated in a largely hydrophobic pocket, lined by the residues Lys101, Ser103, His129, Trp132, Phe133, Tyr154, Tyr157, and Leu171 (Fig. 6*B*). As atDGD2 is only bound to the donor substrate UDP-galactose, we also superimposed the structure with teSPS in complex with UDP and sucrose-6-phosphate (S6P) (27), which represent a GT-B product–bound state. The teSPS product S6P is produced by adding the donor sugar glucose (GLC) to the acceptor sugar fructose-6-phosphate. As noted previously, the UDP of UDP-galactose in atDGD2 binds in the same general area of the donor substrate binding pocket as UDP in teSPS, with small differences in its orientation, mainly for the uracil base (Fig. 6*A*). These slight differences may be representative of what occurs after the donor sugar has been hydrolyzed from its nucleotide. The donor part, GLC, of teSPS product S6P binds in the same position as the donor galactose in UDP-galactose in the structure of atDGD2. As the position of the fructose-6-phosphate lines up nicely with the carboxylic acid of PLM, it may therefore approximate where the galactose of acceptor MGDG binds in atDGD2, and there is indeed ample space for the acceptor substrate. Importantly, this area of the binding pocket has a significant hydrophobic path which would be important for binding of the lipid component of MGDG (Fig. 6*B*).

### X-ray crystal structure in context of previous atDGD2 studies

**Figure 7:**
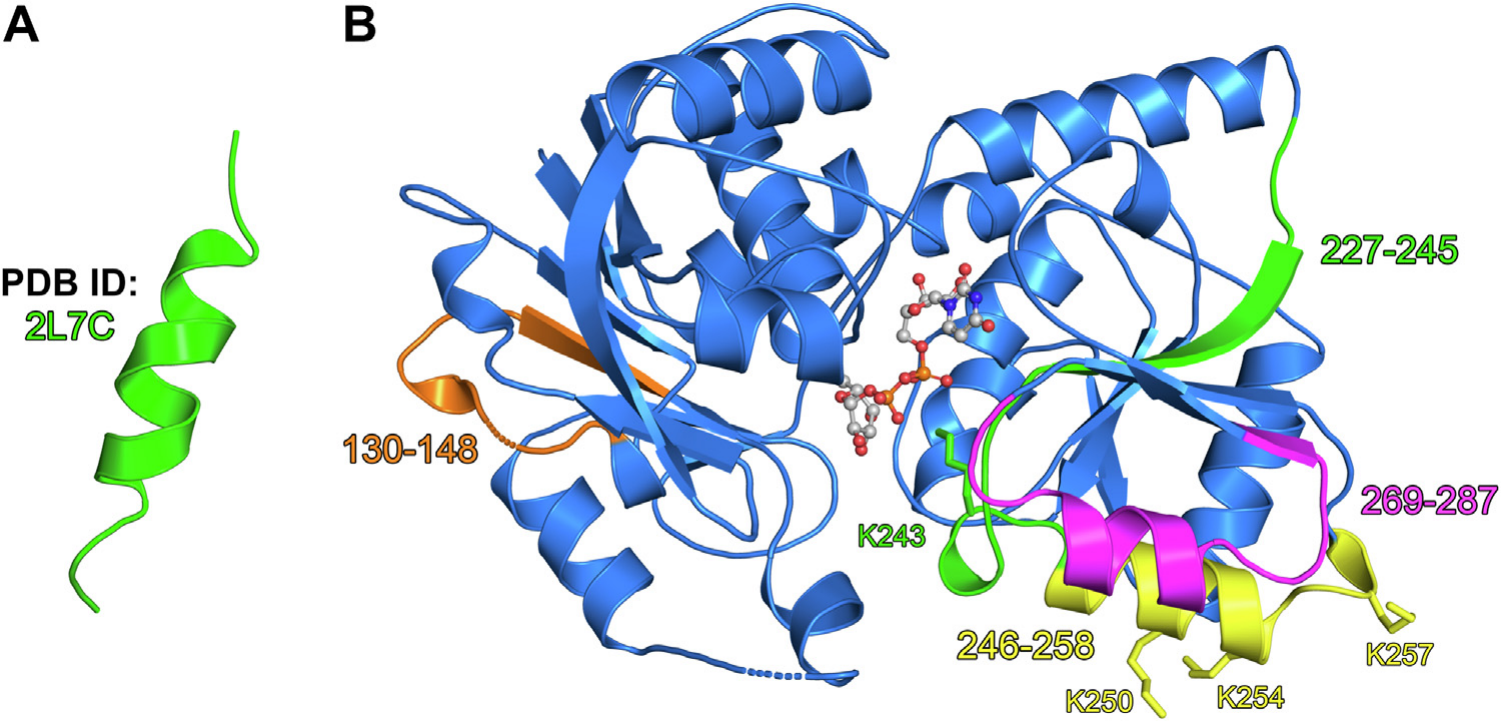
**AtDGD2 structure in context of previous atDGD2 studies.***A*, the structure of region 227 to 245 (PDB ID: 2L7C), which was solved by Solution NMR in dodecylphosphocholine (DPC) micelles (16). *B*, atDGD2 monomer shown as a *blue cartoon*. UDP-galactose is shown as a *ball-and-stick model*. Regions of atDGD2 (amino acids 130–148, 227–245, 246–258, and 269–287) which have been the focus of previous biochemical studies (16, 29) are colored *orange, green, yellow*, and *magenta*, respectively. Four lysine residues (K243, 250, K254, and K257) which were each mutated to alanine in previous studies (15, 29) are shown as *sticks*. Figures were produced with PyMOL (v.2.3.3, Schrödinger).

Prior to this work, two biophysical studies had been performed which investigated the membrane interaction properties of several small segments of atDGD2. In 2011, Szpryngiel *et al.* used circular dichroism and fluorescence spectroscopic techniques to identify possible lipid interacting sites in atDGD2 (16). Two regions of atDGD2 (segments 130–148 from the N-terminal domain and segments 227–245 from the C-terminal domain) were shown to interact with lipids in a charge-dependent manner. Interestingly, the secondary structure of the peptide segments also changed based on the bilayer surface charge. Specifically, when the anionic lipid content of the bilayer was increased, each peptide was transformed into a predominantly α-helical structure. Solution NMR was also used to determine the structure of segments 227 to 245 in dodecylphosphocholine micelles, which also confirmed the helical nature of the segment (Fig. 7*A*) (16). A previous study also indicated that mutating one lysine, K243, to an alanine in this segment completely inactivated the enzyme (15). In the present atDGD2 structure, it is evident that neither segments 130 to 148 nor segments 227 to 245 are α-helical and are instead a combination of β-strand, loop, and 3_10_-helix (Fig. 7*B*). However, it should be noted that part of segments 130 to 148, specifically, residues 133 to 137 are missing in atDGD2 due to structural flexibility.

A further study analyzed the membrane interacting properties of two regions (segments 240–258 and segments 269–287) in atDGD2 using circular dichroism, and mutants in which three lysine residues located in segments 240 to 258 were replaced by alanines were examined. (29). At an anionic lipid concentration of 30 to 40%, segments 240 to 258 were shown to transform from a largely unstructured region into an amphipathic α-helix, which can interact with a bilayer surface in a charge-dependent way. Interestingly, the presence of the acceptor lipid substrate MGDG did not influence the secondary structure of the peptide. Mutation of residues K250, K254, and K257 to alanine caused a significant change in the bilayer interacting properties of segments 240 to 258, indicating that these lysine residues provide important charge dependent interactions. In contrast, segments 269 to 287 were not influenced by the composition of the bilayer and neither the presence of negatively charged lipids nor the acceptor lipid MGDG influenced the secondary structure of the peptide. The authors concluded that segments 240 to 258 and the previously studied segments 227 to 245 and 130 to 139 are good candidates for lipid-sensing switches in atDGD2, which are influenced by the bilayer surface charge, thereby allowing for dynamic events at the membrane. In the present atDGD2 structure, segments 240 to 258 are largely α-helical but also contain loop structure and two 3_10_-helices, and segments 269 to 287 are combination of α-helix, loop, and β-strand, indicating a structural plasticity for these regions (Fig. 7*B*). The ability of a related GT-B, PimA, to adopt two distinct conformations in a crystal in which an entire domain undergoes a dynamic fold-switching indicates that rather large structural rearrangements are indeed possible in these proteins (28, 30).

### Membrane binding kinetics

AtDGD2 needs to become membrane-associated to carry out its enzyme function. An analysis of the location of eight tryptophans in the structure revealed that Trp177 appears to be buried in the membrane (Fig. S8). Because of this, it is feasible to study the membrane interaction, including the lipid substrate interaction, by fluorescence spectroscopy. When any of the tryptophan residues become buried into a hydrophobic environment, or alternatively, if they interact with hydrophobic substrates, their fluorescence yield will increase. The dominating change in signal should thus be due to a change in environment for Trp177. However, we cannot rule out that part of the signal also stems from Trp132, or in fact Trp241 which may interact with lipids when the C-terminal domain comes close to the membrane. This feature was exploited by stopped-flow fluorescence spectroscopy to measure atDGD2’s membrane binding kinetics. The binding was monitored using large unilamellar vesicles (LUVs) that mimic the physicochemical properties of chloroplastic membranes, with a composition that contained 20 mol% of the lipid substrate MGDG together with 70 mol% 1-palmitoyl-2-oleoyl-sn-glycero-3-phosphocholine (POPC) and 10% 1-palmitoyl-2-oleoyl-sn-glycero-3-phospho-(1′-rac-glycerol) (POPG). Since the inner leaflet of the LUVs is not expected to be accessible to the enzyme, the lipid concentration was multiplied by a factor of 0.5 assuming near-equal partition of the lipids in the inner and outer leaflet of the LUVs. Given that on average each lipid head group area is about 0.6 nm^2^, and that the diameter of a LUV is 150 nm, and the LUV molar concentration is about 50 nM at 1 mM lipid concentration. Thus each LUV binds on average four atDGD2 molecules, given that the atDGD2 concentration was about 200 nM. If each atDGD2 interacts with approximately 95 lipids, it occupies an area of around 60 nm^2^, and therefore only a marginal fraction of the LUV surface area is occupied by the enzyme. Analysis of our membrane association model shows that the occluded area contains 105 lipids (Fig. 3*A*), which is quite similar to the value above, based on geometric arguments. The LUV morphology should therefore not be affected by enzyme incorporation and it is safe to assume that the enzyme was able to freely diffuse in the bilayer.

**Figure 8:**
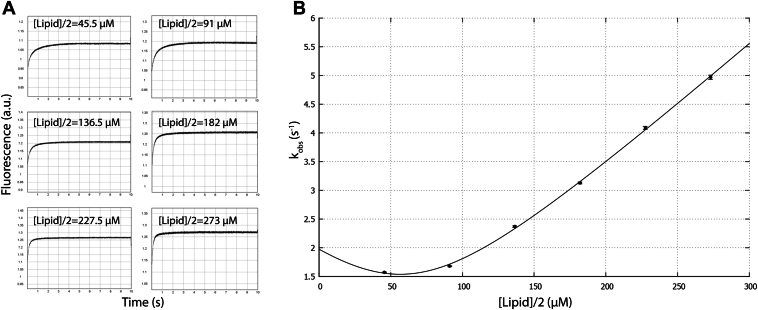
**Lipid binding for atDGD2.***A*, stopped-flow fluorescence traces for atDGD2 in LUVs containing POPC (70 mol%), MGDG (20 mol%), and POPG (10 mol%) at different lipid concentrations as a function of time. The lipid concentration was divided by two to account for inaccessible lipids in the inner leaflet of the LUVs. The extracted *k*_obs_ values were 1.57 ± 0.01, 1.68 ± 0.01, 2.37 ± 0.01, 3.13 ± 0.02, 4.09 ± 0.03, and 4.97 ± 0.04 s^−1^ from lower to higher lipid concentrations. *B*, *k*_*obs*_ from (*A*) as a function of lipid concentration. The data were fitted using Equation 1 described in the Experimental procedures section. atDGD2, digalactosyldiacylglycerol synthase 2; LUV, large unilamellar vesicle; MGDG, monogalactosyldiacylglycerol; POPC, 1-palmitoyl-2-oleoyl-sn-glycero-3-phosphocholine; POPG, 1-palmitoyl-2-oleoyl-sn-glycero-3-phospho-(1′-rac-glycerol).

The change in fluorescence was recorded as a function of time for varying lipid concentrations and from these data the observed rate constants, *k*_*obs*_, were extracted (Fig. 8). The binding parameters could then be extracted by fitting to Equation 1. The fitting gave a *K*_D_ (*k*_off_/*k*_on_) of 180 nM and *k*_on_ = 2.06 μM^−1^ s^−1^, *k*_off_ = 0.37 s^−1^. The result indicated that atDGD2 has medium-to-high affinity for this type of membrane mimetic. Assuming that the membrane association is not specific to certain membrane microdomains, which is reasonable in a LUV model, the number of lipids interacting with each copy of the protein can be estimated by fitting a modified version of the non–pseudo-first-order rate equation. This gave a protein-to-lipid stoichiometry about 1:94.

## Discussion

Herein, we determined the first X-ray crystal structure of atDGD2 in complex with its donor sugar, UDP-galactose, and investigated the enzymes’ membrane interaction kinetics. Structural, biophysical, and biochemical studies have shown that members of the GT-B superfamily can adopt both open and closed forms (31, 32). It has been suggested that binding of the nucleotide sugar donor triggers a closure movement involving the rotation of the N- and C-terminal domains, which is needed to bring together critical residues from each domain to form a functional active site (25, 26, 27). In the open conformation, the catalytic interface is not formed and represents a structural state of precatalysis or postcatalysis (33). Depending on the GT-B enzyme, the conformational change between open and closed states can be subtle, such as what has been observed for bacterial VldE or atMGD1, where the difference in conformation primarily involves active site loops (18, 34). For other GTs, such as MshA, this conformational change can involve a major reorientation of the N- and C-terminal domains, in addition to rearrangements of active site loops (35). It has also been demonstrated that in some cases, like in PimA and WaaG, multiple conformations corresponding to open and closed conformations exist in equilibrium, and that populations and conformational dynamics are important in determining enzyme function (36, 37). The atDGD2-UDP-galactose structure most likely represents a stabilized closed form of the enzyme. As we were unable to crystallize atDGD2 without ligands, it is unclear what structural changes occur to the apo enzyme upon nucleotide donor sugar binding, although this clearly hints to the possibility that atDGD2 also adopts several conformational states. Furthermore, as we were unable to obtain a structure in complex with MGDG, it is not possible to say what structural changes occur upon acceptor substrate binding. During atDGD2 refinement, we noted two areas of missing electron density in the N-terminal domain, where the surrounding regions had the highest *B*-factors in the overall structure, which we attributed to structural flexibility. This may be due to the absence of MGDG in the structure, as one of the segments of missing density is in close proximity to the expected acceptor substrate binding site. As we were unable to obtain crystals in the presence of both UDP-galactose and MGDG, it is possible that the acceptor actually has a destabilizing effect, which would also be in agreement with the presence of several conformations. This has been observed for PimA, where binding of the sugar donor GDP-mannose triggers a stabilized closed form of the enzyme, whereas the phospholipid acceptor substrate phosphatidylinositol has a destabilizing effect. As this was also observed in the presence of GDP mannose, the authors concluded that phosphatidylinositol further modifies the closed conformation to a more relaxed enzyme–ligand complex (31). This was also shown for atMGD1, where the enzyme was unable to be crystallized in complex with any lipid acceptor substrates (18). However, it should be noted that this is not the case for all GT-B enzymes, as well diffracting crystals of teSPS were only obtainable in the presence of both a nucleotide donor sugar and the enzyme’s acceptor substrate (27).

The majority of GTs utilizes donors containing diphosphate leaving groups, such as UDP and GDP (30, 38, 39). To date, two main structural superfamilies have been identified, named GT-A and GT-B, with very low amino acid sequence identity between enzymes within the same family (14, 40, 41). AtDGD2 is most structurally similar to other GT-B superfamily members, with the C-terminal domains of the enzymes having the highest structural similarity. This may be due to the fact that it is predominantly residues from the C-terminal domain which are required to bind the nucleotide sugar donor, which are significantly less diverse than acceptor substrates used by GTs. Previous structural studies have shown that it is primarily residues from the N-terminal domain in GT-B fold enzymes that are responsible for coordinating the acceptor substrate (38, 42, 43). This may explain the greater variation observed for the N-terminal domains of atDGD2 and structurally similar GTs. While the active site of atDGD2 differs significantly to related GTs, there were four amino acids that were entirely conserved (Gly22, His151, Lys243, and Glu321). There are no mutational studies for Gly22 in related GT-B enzymes. However, the strong conservation is likely due to glycine lacking a sidechain, and thus anything larger would directly clash with either the phosphate group and/or sugar of the nucleotide sugar donor substrate, which could negatively impact its positioning in the active site. While no mutagenesis studies have been performed in related GT-B proteins for the equivalent lysine, previous atDGD2 studies have shown that mutating Lys243 to alanine abolishes enzyme activity (15). We speculate that in addition to its critical role in correctly positioning the phosphate group of the donor substrate, this lysine may also be important for stabilizing the transition state and enhancing the departure of UDP during catalysis. Mutagenesis studies have been performed for the residues equivalent to Glu321 and His151, which have provided insights into their importance for GT activity. Mutation of this glutamate to alanine in teSPS inactivated the enzyme, suggesting that optimal positioning of the nucleotide’s ribose moiety is critical for proper enzymatic function (27). Mutation of the equivalent histidine in teSPS, saBshA, and bsBshA resulted in catalytically dead enzymes, and this histidine was therefore suggested to play an important role in the enzymes’ catalytic mechanism (25, 26, 27). These GTs are proposed to operate via a substrate-assisted substitution nucleophilic internal (S_N_i) mechanism involving a short-lived oxocarbenium-like intermediate, where the UDP leaving group departs from the same face of the sugar as the nucleophilic hydroxyl from the acceptor substrate attacks (44). In the product-bound teSPS structure, the conserved histidine together with the phosphate group of UDP was shown to hydrogen bond with the GLC portion of the S6P product (27). This interaction results in the four hydroxyl groups of the sugar becoming partially negatively charged, which is suggested to promote the formation of the oxocarbenium ion required for catalysis. Interestingly, while most GT-A GTs require divalent metal ions for activity, members of the GT-B fold do not need metal ions for catalysis (17, 45, 46). Without metal ions to anchor the phosphate group of the nucleotide sugar donor in the active site, it is proposed that in GT-B GTs, the negatively charged pyrophosphate group is instead stabilized through α-helix dipole effects, in addition to the positively charged sidechains of amino acids such as arginine and/or lysine (47).

Interestingly, atDGD2 was less structurally similar to atMGD1, which catalyzes the preceding step of lipid glycosylation in *A. thaliana*. Despite atDGD2 and atMGD1 both using UDP-galactose as the nucleotide sugar donor, and having relatively more similar acceptor substrates compared to the other GTs, the enzymes exhibited very different donor substrate binding hydrogen bond networks. However, the aforementioned conserved glutamate is also conserved in atMGD1, further validating its importance. As shown for structurally related GT-B enzymes, the His155A mutant (His151 in atDGD2) was completely inactive (18). This histidine is critical for the reaction mechanism of atMGD1, where it is proposed to aid in the deprotonation of the nucleophile OH group of the DAG acceptor substrate. This amino acid does not superimpose well when the atDGD2 and atMGD1 structures are compared, due to significant movements for certain loops in the active site. Interestingly, while we were unable to crystallize atDGD2 in the absence of ligands, an apo structure of atMGD1 was solved in addition to the UDP-bound structure (18). Comparison of these structures shows no major conformational changes for the core structure of the enzyme; however, there are two loop regions in the active site which shift significantly upon UDP binding. The differences in active site binding loops between apo MGD1, UDP-bound atMGD1, and UDP-galactose–bound atDGD2 may indicate that the structures represent different precatalytic and postcatalytic states.

AtDGD2 has the challenging task of binding to both water-soluble and highly hydrophobic substrates simultaneously, and its membrane binding properties are crucial for efficient catalysis. Monotopic GTs can interact with biological membranes through anchoring via one or more amphipathic α-helices, which often occurs in combination with additional electrostatic interactions provided by cationic amino acids such as lysine or arginine. The amino acid tryptophan is also known to be enriched in bilayer interface regions and is important for membrane interaction in both monotopic and transmembrane proteins (48, 49, 50). Tryptophans are abundant in atDGD2, particularly in the N-terminal domain, which contains seven of the enzymes’ eight Trp residues (Fig. S8). Our membrane interaction model revealed that Trp177 is buried in the membrane (Fig. 3*A*). Trp241, the only Trp in the C-terminal domain, may be involved in membrane anchoring of the C-terminal domain, although previous studies have demonstrated that electrostatic interactions are important for membrane interactions (51). Trp132 lines the hydrophobic cavity of the proposed MGDG binding site and Trp19 is in close proximity to the UDP-galactose binding site and are thus likely to be involved in lipid substrate interactions. At least four tryptophans—Trp48, Trp77, Trp139, and Trp380—are surface exposed and are located in the region of the N-terminal domain associated with the highest *B*-factors in the atDGD2 structure, which includes the helix α5. Previous studies have shown all tryptophans, including the surface exposed ones, are extremely important for atDGD2 activity. Although it was not determined what the cause of loss of activity was, it was sufficient to mutate any one of them to phenylalanine or alanine to reduce the enzyme activity to almost zero (15). Taken together with our structural information, this further supports that the region of atDGD2 containing Trp177 in the N-terminal domain is highly important for modulating membrane interaction.

The binding kinetics gave a quantitative insight of atDGD2’s membrane association. The activity assay clearly indicated that atDGD2 is nearly completely membrane-associated (*K*_D_ = 180 nM) under the conditions used (POPC/POPG/MGDG lipids). It is interesting to note that a wide range of dissociation constants have been found for other GT-B enzymes. For *Arabidopsis laidlawii* GT MGS, K_D_s in the nM to pM range have been reported (52), while for the *Mycobacterium tuberculosis* PimA, weaker binding on the order of 100 μM were observed (53). Similar results were also seen for *E. coli* WaaG (54). These results imply that these enzymes are finely tuned to interact with membranes in different ways. Although the binding appears to be relatively strong for atDGD2, the *k*_on_ (2.06 μM^−1^ s^−1^) and *k*_off_ (0.37 s^−1^) parameters indicate that the equilibrium is highly dynamic. This suggests that for each enzymatic cycle, the enzyme may undergo several cycles of adsorbing and desorbing to the membrane before a catalysis cycle is accomplished. The composition of the bilayer itself is also known to be important for modulating atDGD2 activity, as exposure to negatively charged lipids has been shown to increase the activity of the enzyme (15, 17). Previous biochemical studies of atDGD2 identified three segments (residues 130–148, 227–245, and 240–250) which change their secondary structure to being predominantly α-helical when the anionic charge is increased (16, 29). In our atDGD2 structure, regions 227 to 245 which contain the catalytically relevant lysine Lys243 are immediately followed by an amphipathic α-helix which is rich in lysine residues. It has been shown that mutation of Lys250, Lys254, and Lys257 to alanine in segment 246 to 258 negatively impacts the atDGD2 activity (29), which may indicate that these positively lysines are important for interacting with negatively charged phospholipids in the bilayer. Interestingly, segments 130 to 148 and 227 to 245 are not α-helical in our atDGD2 structure, which may be due to the fact that atDGD2 was purified in the presence of the detergent DDM, which is nonionic and therefore uncharged. Interestingly, secondary structure “fold-switching” has previously been reported for the monotopic membrane associated GT *M tuberculosis* PimA. In these investigations, PimA demonstrated remarkable structural plasticity, particularly for segments of the N-terminal domain which in the presence of anionic phospholipids in the bilayer undergo dramatic secondary structure changes, including β-strand to α-helix and α-helix to β-strand transitions (28, 31, 32). Evidently, such secondary structure reshuffling is important for modulating protein function, allowing these membrane-associated GTs to adjust their activity in response to the composition of the bilayer, which in turn ensures very rapid capture and utilization of membrane lipid acceptor substrates.

In summary, we present the first structure of atDGD2 in complex with the donor substrate UDP-galactose, and membrane binding kinetic studies in LUVs. AtDGD2 is most structurally similar to functionally unrelated GT-Bs. In spite of significant differences in donor substrate binding, our analysis identified four important amino acids which are entirely conserved between these proteins. Interestingly, atDGD2 shared less structural similarity to atMGD1, which catalyzes the preceding step of lipid glycosylation in *A. thaliana*. Our membrane binding studies showed atDGD2 is strongly associated with the membrane, but also demonstrated that its membrane association is highly dynamic. This information in the context of our atDGD2 structure and previously performed biochemical studies highlights regions of atDGD2 with a high degree of structural plasticity, which may allow the enzyme to quickly modulate its activity based on the lipid bilayer environment.

## Experimental procedures

### Protein expression and purification

A plasmid DNA construct encoding truncated *A. thaliana* DGD2 (atDGD2Δ401-473) was expressed and purified as described previously (17). In brief, atDGD2Δ401-473 was overexpressed in *E. coli* BL21-AI cells (Thermo Fisher Scientific) in TB medium supplemented with 0.2% (*w*/*v*) L-arabinose and 50 μg/ml kanamycin. Following induction by addition of IPTG, the cells were grown at 18 °C for 24 h. Cells were harvested, lysed, and then purified by immobilized metal affinity chromatography, followed by size-exclusion chromatography. Fractions containing atDGD2 were pooled and concentrated to 10 mg/ml. The protein purity was assessed using SDS-PAGE (Fig. S9). SeMet-atDGD2 was produced as per the native enzyme, with the exception that cells were grown in M9 media. Amino acid mix was added at an *A*_600 nm_ of 0.6, 20 min prior to induction.

### Crystallization and structure determination

Purified SeMet-atDGD2 (10 mg/ml) was preincubated with 5 mM UDP-galactose prior to setting sitting-drop crystallization plates. Crystals were obtained in the Morpheus screen (Molecular Dimensions) D12 condition containing 0.1 M bicine/Trizma base pH 8.5, 12.5% (*w*/*v*) PEG 1000, 12.5% (*w*/*v*) PEG 3350, 12.5% (*v*/*v*) 2-methyl-2,4-pentanediol, 0.02 M 1,3-propanediol, 0.02 M 1,4-butanediol, 0.02 M 1,6-hexanediol, 0.02 M 1-butanol, and 0.02 M 2-propanol. Crystals were frozen in liquid nitrogen without additional cryoprotectant. X-ray diffraction data was collected at the PXII beamline of the Swiss Light Source Synchrotron. Prior to data collection a fluorescence scan was performed and a weak Se peak was found, after which the wavelength of the beam was adjusted to 0.98 Å. Data were processed using DIALS (55) and phases were solved using AutoSol (56). The structure was built using Coot (57) and refined in REFMAC (58). Data collection and refinement statistics are presented in Table S1. The coordinates and structure factors for atDGD2-UDP-galactose were deposited in the PDB under the accession code 8P6S.

### Stopped-flow fluorescence spectroscopy

Purified atDGD2 and LUVs were used for assessing binding affinity. The lipids used for producing LUVs MGDG, POPC, and POPG were obtained from Avanti Polar Lipids, Inc. LUVs were prepared by dissolving lipids in chloroform with a molar ratio of 70% POPC (mol/mol), 20% MGDG, and 10% POPG and mixed. The solvent was evaporated under a stream of nitrogen and resuspended in in 50 mM sodium phosphate buffer pH 7.4 and 200 mM NaCl by vortexing for 30 min to form multilamellar vesicles. The solution was extruded through filters of 100 nm in diameter to obtain a monodisperse solution of LUVs. The LUVs were rapidly mixed with purified atDGD2 for monitoring the fluorescence yield changes. The increase of fluorescence yield upon rapid mixing of the protein and LUVs was recorded using a SX20 stopped-flow spectrometer (Applied Photophysics). The experimental temperature was set to 25 °C and stabilized by a water bath thermostat. The excitation wavelength was 280 nm and fluorescence emission was detected after passage through a 320 nm long pass filter. The protein sample and LUV sample was mixed at a 1:10 ratio (20 μl protein sample with 200 μl LUV sample). After mixing, the atDGD2 concentration was about 0.78 μM, and lipid concentrations were 45.5, 91, 136.5, 182, 227.5, and 273 μM. At each LUV concentration, five traces were recorded and averaged and fitted to a monoexponential function to extract the observed rate constant, *k*_*obs*_. The observed rate constant extraction was done by using PRO-DATA SX software, Applied Photophysics (https://www.photophysics.com/media/gqectt12/sx-pro-data-upgrade-2016.pdf). The observed rate constants were fitted to the modified non-pseudo first order equation (Eq 1):

(1) $$k_{obs}=\sqrt{\left(k_{on}^{2}\times {\left(\left[E\right]-C_{1}\times \left[L\right]\right)}^{2}+k_{off}^{2}+2\times k_{on}\times k_{off}\times \left(\left[E\right]+C_{1}\times \left[L\right]\right)\right)}$$

where [E] is the protein concentration, [L] is the lipid concentration, *C*_1_ is a factor related to the stoichiometry, and *k*_on_ and *k*_off_ are the association and dissociation rate constant.

## Data availability

The protein structure presented in this paper has been deposited in the PDB under the accession code 8P6S. All remaining data are contained within the article.

## Supporting information

This article contains supporting information (60, 61).

## Conflict of interests

The authors declare that they have no conflicts of interest with the contents of this article.
