# Supplementary material for: Structure and membrane interactions of Arabidopsis thaliana DGD2, a glycosyltransferase in the chloroplast membrane
Source: J Biol Chem. 2025 Mar 20;301(5):108431. doi: 10.1016/j.jbc.2025.108431 (PMC12022483; doi:10.1016/j.jbc.2025.108431)
Supplement: Supporting information [file mmc1.docx]

**Structure and membrane interactions of *Arabidopsis thaliana* DGD2, a glycosyltransferase in the chloroplast membrane**

*Emma Scaletti Hutchinson, Markel Martínez-Carranza^1^, Biao Fu, Lena Mäler^*^ and Pål Stenmark^*^*

Department of Biochemistry and Biophysics, Stockholm University, SE-106 91 Stockholm, Sweden

^1^Current address: Department of Structural Biology and Chemistry, Pasteur Institute, 75017 Paris, France

^*^Corresponding author. Correspondence and requests for materials should be addressed to Prof. Lena Mäler or Prof. Pål Stenmark, Department of Biochemistry and Biophysics, Stockholm University, SE-106 91 Stockholm, Sweden, E-mail: lena.maler@dbb.su.se or stenmark@dbb.su.se.


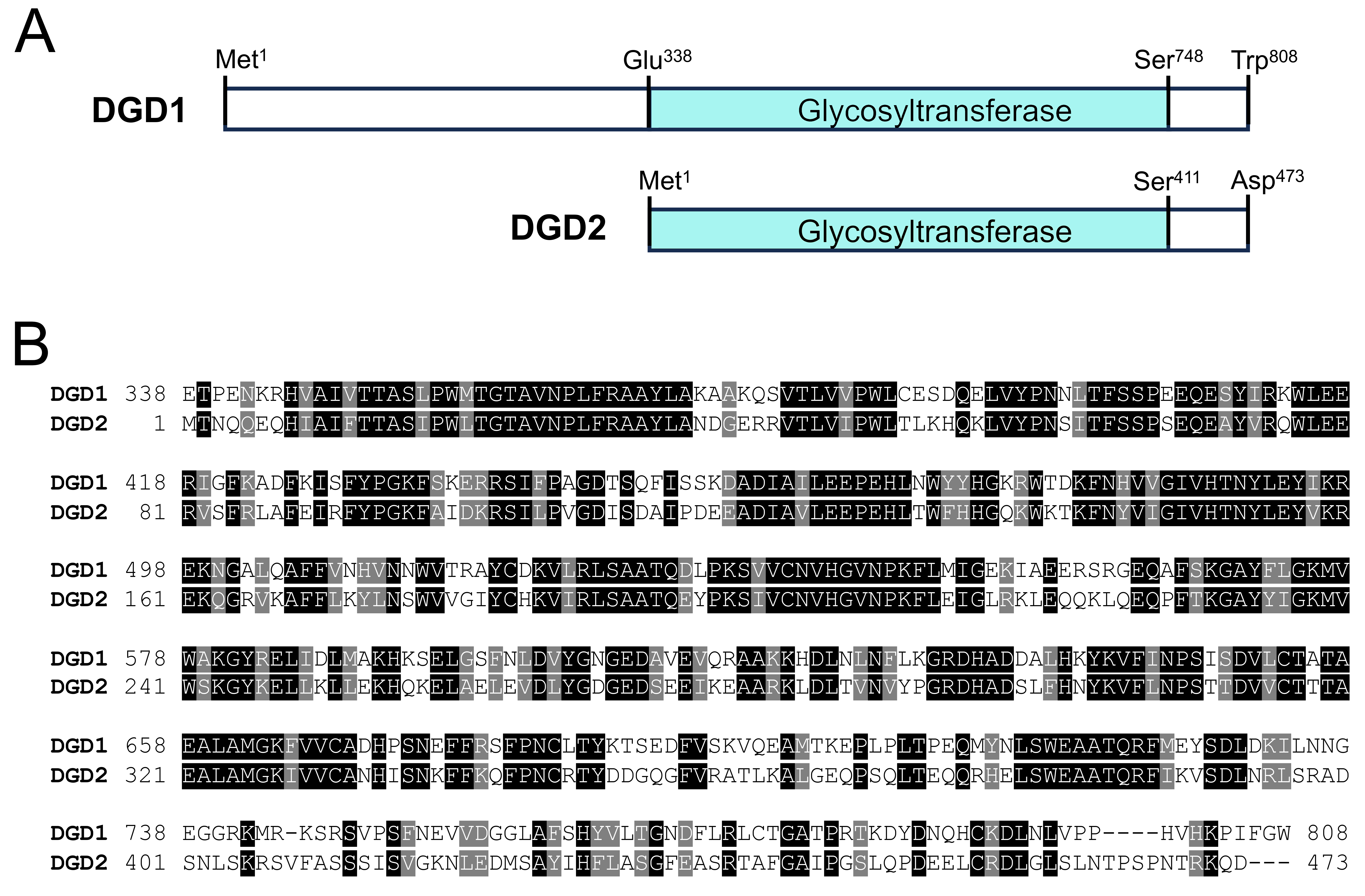


**Figure S1. Comparison of atDGD1 and atDGD2 proteins. (A)** Overall domain structure of atDGD1 (UniProt: Q9S7D1) and atDGD2 (UniProt: Q8W1S1), highlighting the position of the glycosyltransferase domain responsible for hydrolyzing UDP-galactose. **(B)** Amino acid sequence alignment of the glycosyltransferase domain of atDGD1 and atDGD2 performed using Clustal Omega through the EBI webserver. The resulting alignment is colored according to sequence similarity using BOXSHADE. Identical residues are shaded black, while grey shading indicates amino acids with conserved physicochemical properties. The amino acid sequence identity between these glycosyltransferase domains is 54.8 %.

**
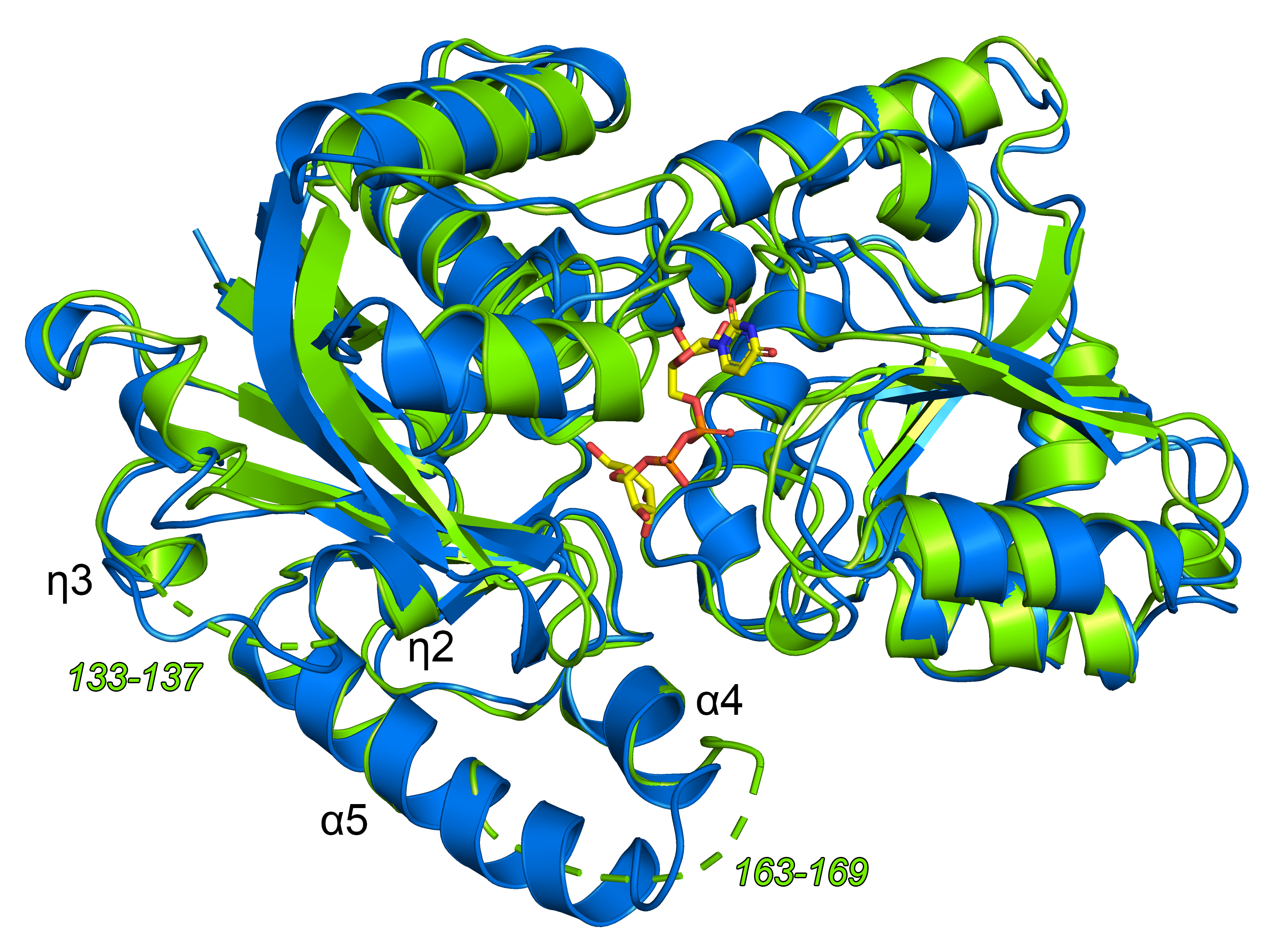
**

**Figure S2. Comparison of atDGD2 with computationally generated model.** An Alphafold model was produced using the Alphafold webserver (<https://alphafold.ebi.ac.uk>) and the amino acid sequence of atDGD2-Δ401-473 (UniProt: Q8W1S1) as the input. Individual monomers are shown as cartoon representations colored green (atDGD2, this study) or blue (atDGD2, Alphafold model). Cα-atom superposition of the two structures is associated with an RMSD of 1.48 Å. Two areas of missing density (residues 133-137 and 163-169) in the atDGD2 X-ray crystal structure are highlighted. Relevant secondary structure elements in this regions including 3_10_-helices 2 and 3 (η2 and η3) and α-helices 4 and 5 (α4 and α5) are indicated. UDP-galactose from the experimentally determined atDGD2 structure is depicted as a stick model; C atoms are colored yellow, O atoms red, N atoms blue and P atoms orange. Figure were produced with PyMOL (v.2.3.3, Schrödinger).


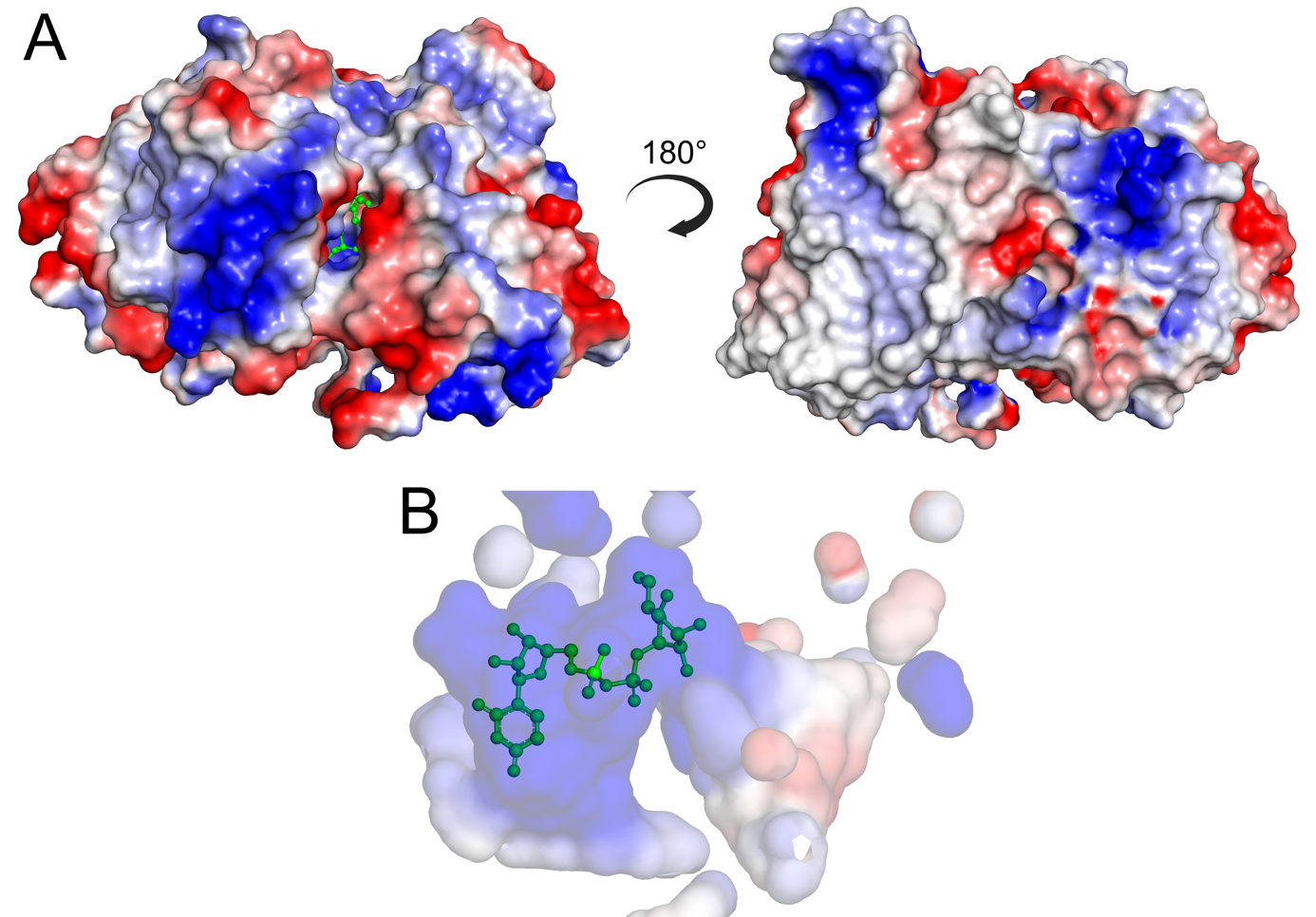


**Figure S3. Electrostatic potential of atDGD2. (A)** Protein surface and **(B)** active site binding pocket of atDGD2 colored according to electrostatic potential. The donor sugar substrate UDP-galactose from atDGD2 is shown as a green ball and stick model. The electrostatic potential was calculated with APBS (1) in the range- 5 kT (red, positive potential) to + 5 kT (blue, negative potential). Figures were produced with PyMOL (v.2.3.3, Schrödinger).


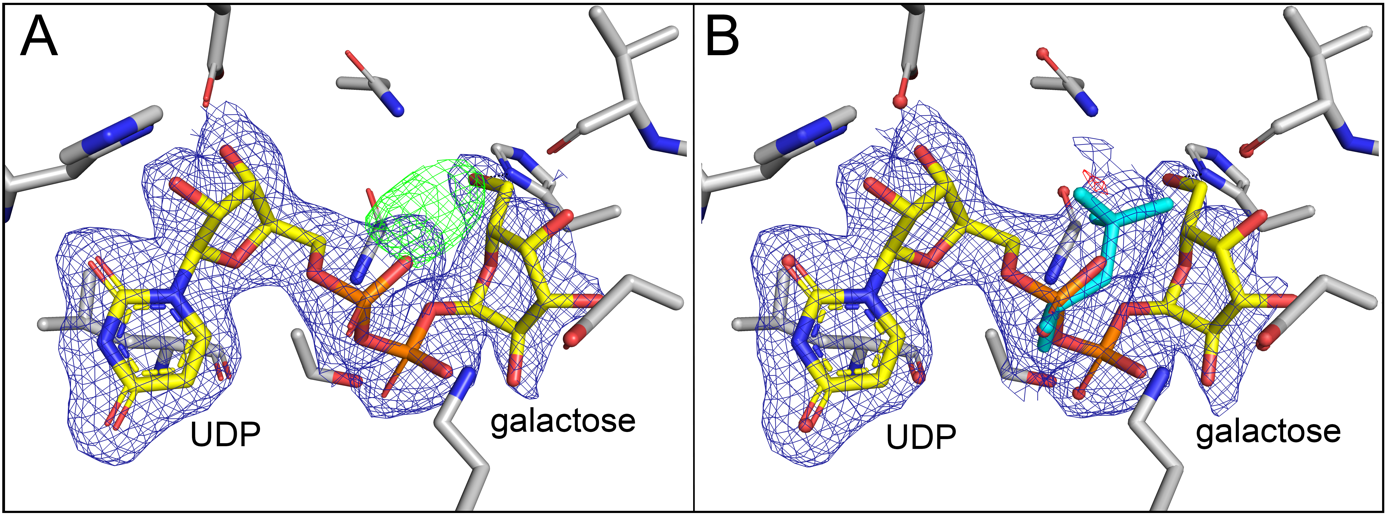


**Figure S4. Modelling of additional positive density peak in the atDGD2 active site. (A)** Electron density maps following refinement when UDP-galactose only is built into the donor substrate binding pocket. UDP-galactose is depicted as a stick model; C atoms are colored yellow, O atoms red, N atoms blue and P atoms orange. Amino acids involved in ligand coordination are shown as sticks. **(B)** Electron density maps following refinement when both UDP-galactose and UDP are built into the active site. For clarity, only the additional phosphates of UDP are shown in cyan, as the uridine portion of the ligand adopts an identical position to the uridine moiety of UDP-galactose. The 2*F*_o_−*F*_c_ electron density maps in each panel are contoured at 1.0 *σ* (blue), and the *F*_o_−*F*_c_ electron density maps are contoured at +3.5 *σ* (green) and -3.5 *σ* (red). Figures were produced with PyMOL (v.2.3.3, Schrödinger).

**
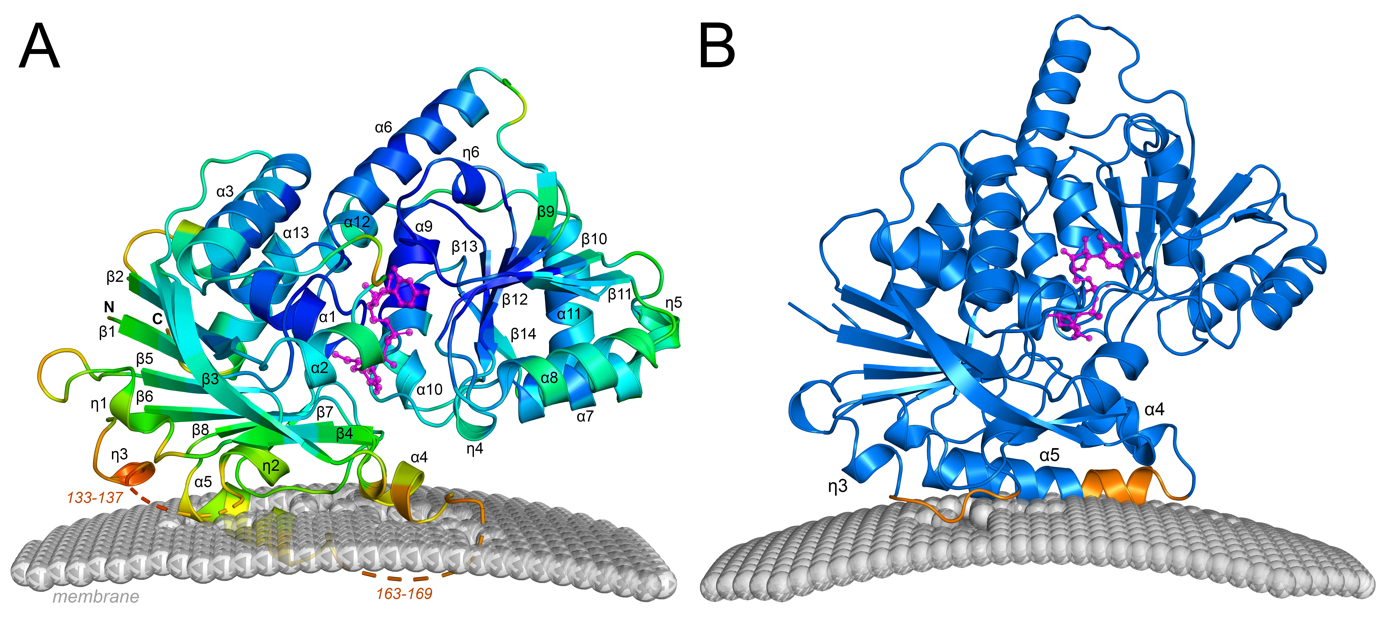
**

**Figure S5. Interaction of atDGD2-UDP-galactose and atDGD2 Alphafold model with the chloroplast outer membrane.** **(A)** Membrane interaction model created using the Orientation of proteins in membranes server (<https://opm.phar.umich.edu/ppm_server3_cgopm/>). The atDGD2 monomer is shown as a cartoon with secondary structure annotation, colored according to *B*-factor. The *B*-factors are depicted on the structure in dark blue (lowest *B*-factor) through to red (highest *B*-factor). Two segments of missing density (residues 133-137 and 163-169) from the atDGD2 structure are indicated. **(B)** Membrane interaction model for the atDGD2 Alphafold model, which was produced using the Alphafold webserver (<https://alphafold.ebi.ac.uk>) and the amino acid sequence of atDGD2-Δ401-473 (UniProt: Q8W1S1) as input. The atDGD2 Alphafold model is shown as a cartoon representation colored blue. The two segments of missing density from atDGD2-UDP-galactose (aa133-137 and 163-169) are colored orange. In both panels **A** and **B**, UDP-galactose from the experimentally determined atDGD2 structure is shown as a magenta ball‑and‑stick model. Figure produced with PyMOL (v.2.3.3, Schrödinger).

*
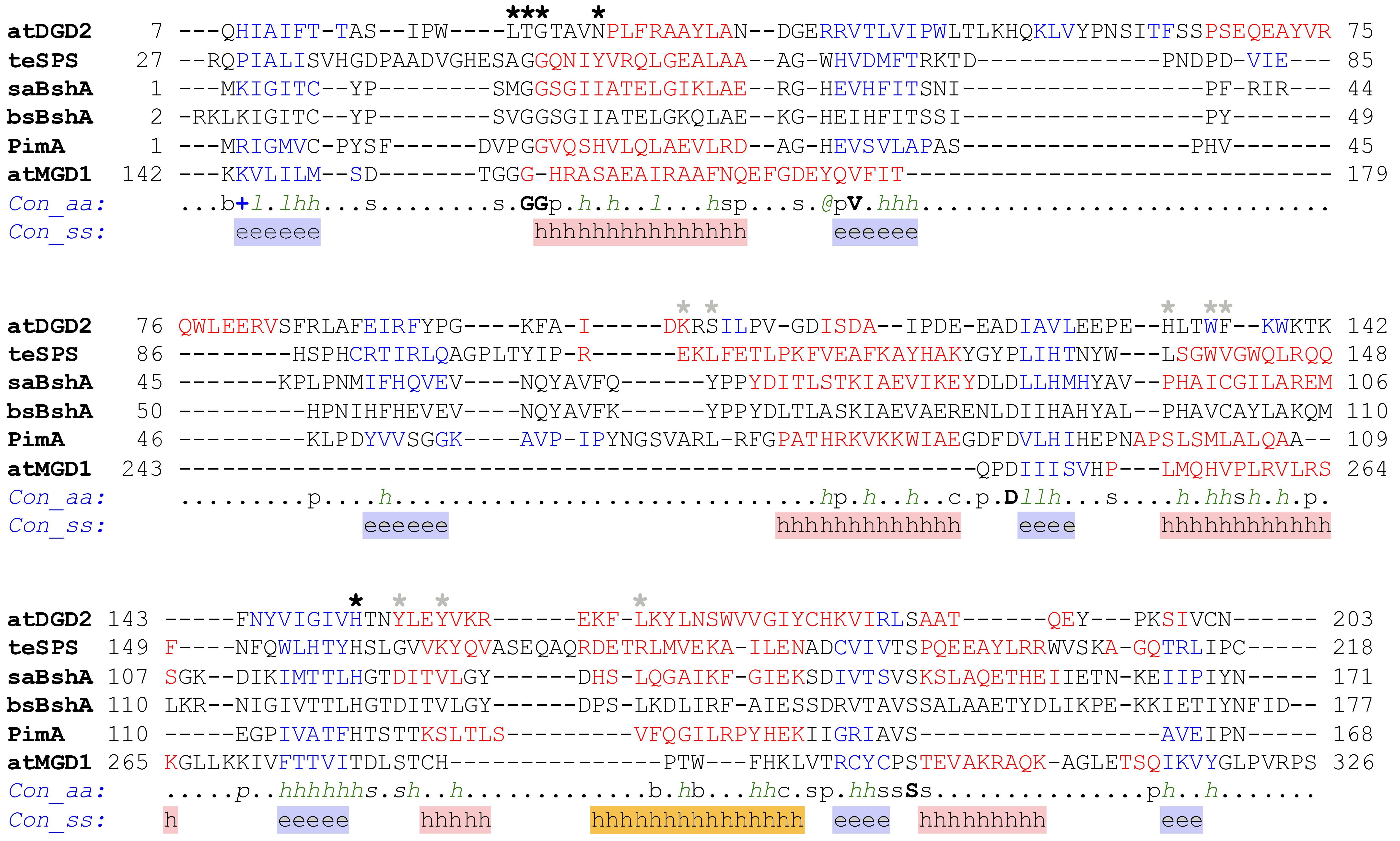
*

**Figure S6. Structure-based sequence alignment of atDGD2 N-terminal domain with structurally related glycosyltransferases.** The PROMALS3D webserver (2) was used to create the alignment using the N-terminal domains (residue ranges listed in **Table S3**) of atDGD2 (this work), *T. elongatus* Sucrose-phosphate synthase (teSPS, PDB: 6kih), *S. aureus* BshA (saBshA, PDB: 6d9t), *B. subtilis* glycosyltransferase BshA (bsBshA, PDB: 5d00), *M. smegmatis* phosphatidyl mannosyltransferase (PimA, PDB: 4n9w) and *A. thaliana* MGD1 (atMGD1, PDB: 4x1t). *Con_ss*: consensus secondary structure predictions (h: α-helix, shaded pink and b: β-strand, shaded lilac). *Con_aa*: consensus amino acids. If the weighted frequency of a particular type of amino acid is above a certain threshold, the consensus symbol of that type is shown. Symbols are as follows: conserved amino acids (bold and uppercase letters), aliphatic residues I, V, L (*l*), aromatic residues Y, H, W, F (@), hydrophobic residues W, E, Y, M, L, I, V, A, C, T, F (*h*), alcohol residues S, T (o), polar residues D, E, H, K, N, Q, R, S, T (p), tiny residues A, G, C, S (t), small residues A, G, C, S, V, N, D, T, P (s), bulky residues E, F, I, K, L, M, Q, R, W, Y (b), positively charged residues K, R, H (+), negatively charged residues D, E (-) and charged residues D, E, K, R, H (c). Sequences are colored according to PSIPRED secondary structure predictions (red: α-helix, blue: β-strand). Black asterisks indicate amino acids from atDGD2 involved in UDP-galactose binding, and grey asterisks indicate residues proposed to interact with the lipid acceptor substrate MGDG. The important membrane interacting helix α5 in atDGD2 is highlighted yellow.

*
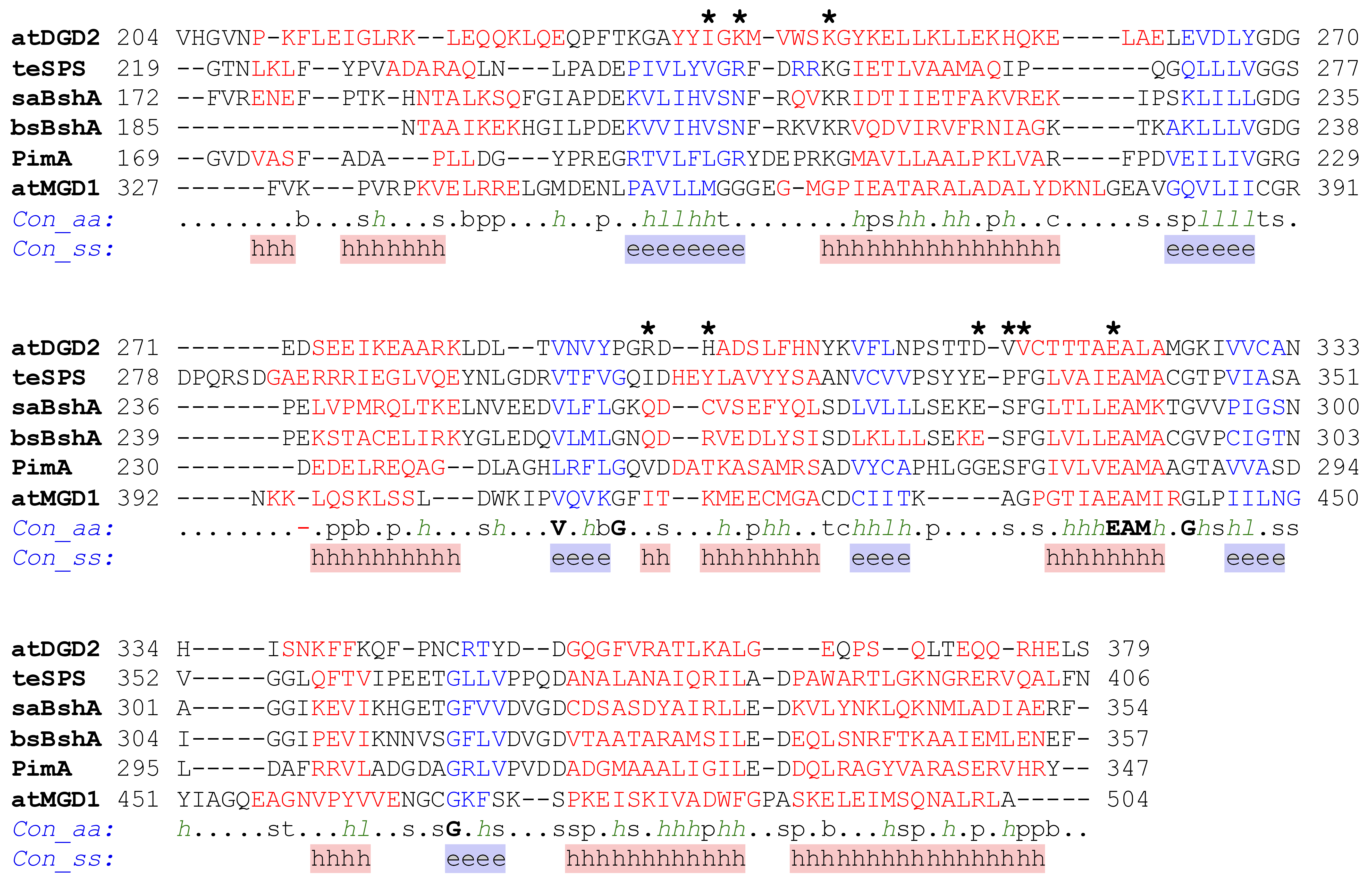
*

**Figure S7. Structure-based sequence alignment of atDGD2 C-terminal domain with structurally related glycosyltransferases.** The PROMALS3D webserver (2) was used to create the alignment using the C-terminal domains (residue ranges listed in **Table S3**) of atDGD2, *T. elongatus* Sucrose-phosphate synthase (teSPS, PDB: 6kih), *S. aureus* BshA (saBshA, PDB: 6d9t), *B. subtilis* glycosyltransferase BshA (bsBshA, PDB: 5d00), *M. smegmatis* phosphatidyl mannosyltransferase (PimA, PDB: 4n9w) and *A. thaliana* MGD1 (atMGD1, PDB: 4x1t). *Con_ss*: consensus secondary structure (h: α-helix, shaded pink and b: β-strand, shaded lilac). *Con_aa*: consensus amino acids. If the weighted frequency of a particular type of amino acid is above a certain threshold, the consensus symbol of that type is shown. Symbols are as follows: conserved amino acids (bold and uppercase letters), aliphatic residues I, V, L (*l*), aromatic residues Y, H, W, F (@), hydrophobic residues W, E, Y, M, L, I, V, A, C, T, F (*h*), alcohol residues S, T (o), polar residues D, E, H, K, N, Q, R, S, T (p), tiny residues A, G, C, S (t), small residues A, G, C, S, V, N, D, T, P (s), bulky residues E, F, I, K, L, M, Q, R, W, Y (b), positively charged residues K, R, H (+), negatively charged residues D, E (-) and charged residues D, E, K, R, H (c). Sequences are colored according to PSIPRED secondary structure predictions (red: α-helix, blue: β-strand). Black asterisks indicate amino acids from atDGD2 involved in UDP-galactose binding. The important membrane interacting helix α5 in atDGD2 is highlighted yellow.

*
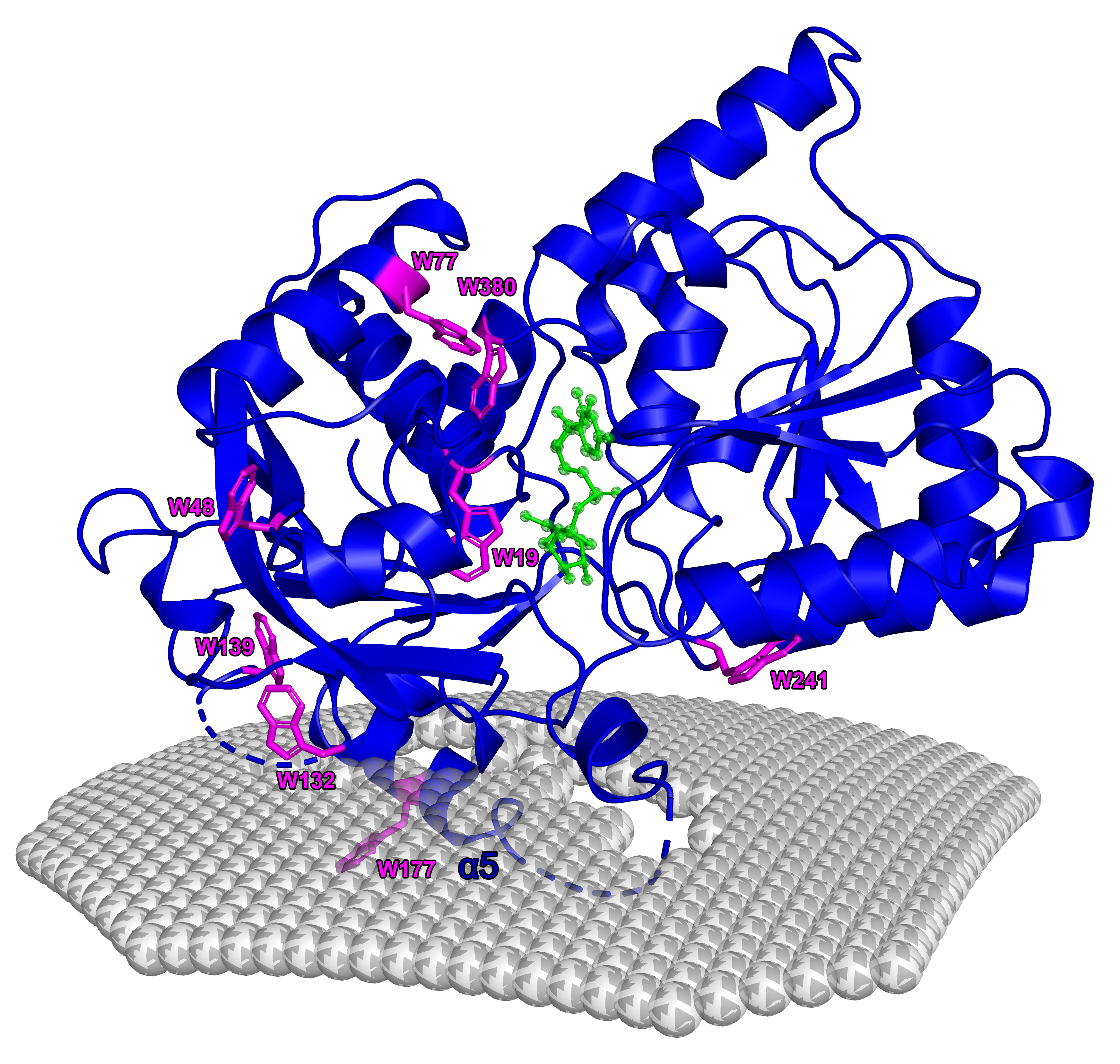
*

**Figure S8. AtDGD2-UDP-galactose chloroplast outer membrane model highlighting Tryptophan residues.** A Membrane interaction model was created using the Orientation of proteins in membranes server (<https://opm.phar.umich.edu/ppm_server3_cgopm/>). The atDGD2 monomer is shown as a dark blue cartoon representation. The atDGD2 structure contains 8 Tryptophan residues (W19, W48, W77, W132, W139, W177, W241 and W380) which are shown as magenta sticks. Tryptophan W177 located on α-helix 5 (α5) is buried within the membrane. UDP-galactose is shown as a green ball‑and‑stick model. Figure produced with PyMOL (v.2.3.3, Schrödinger).

*
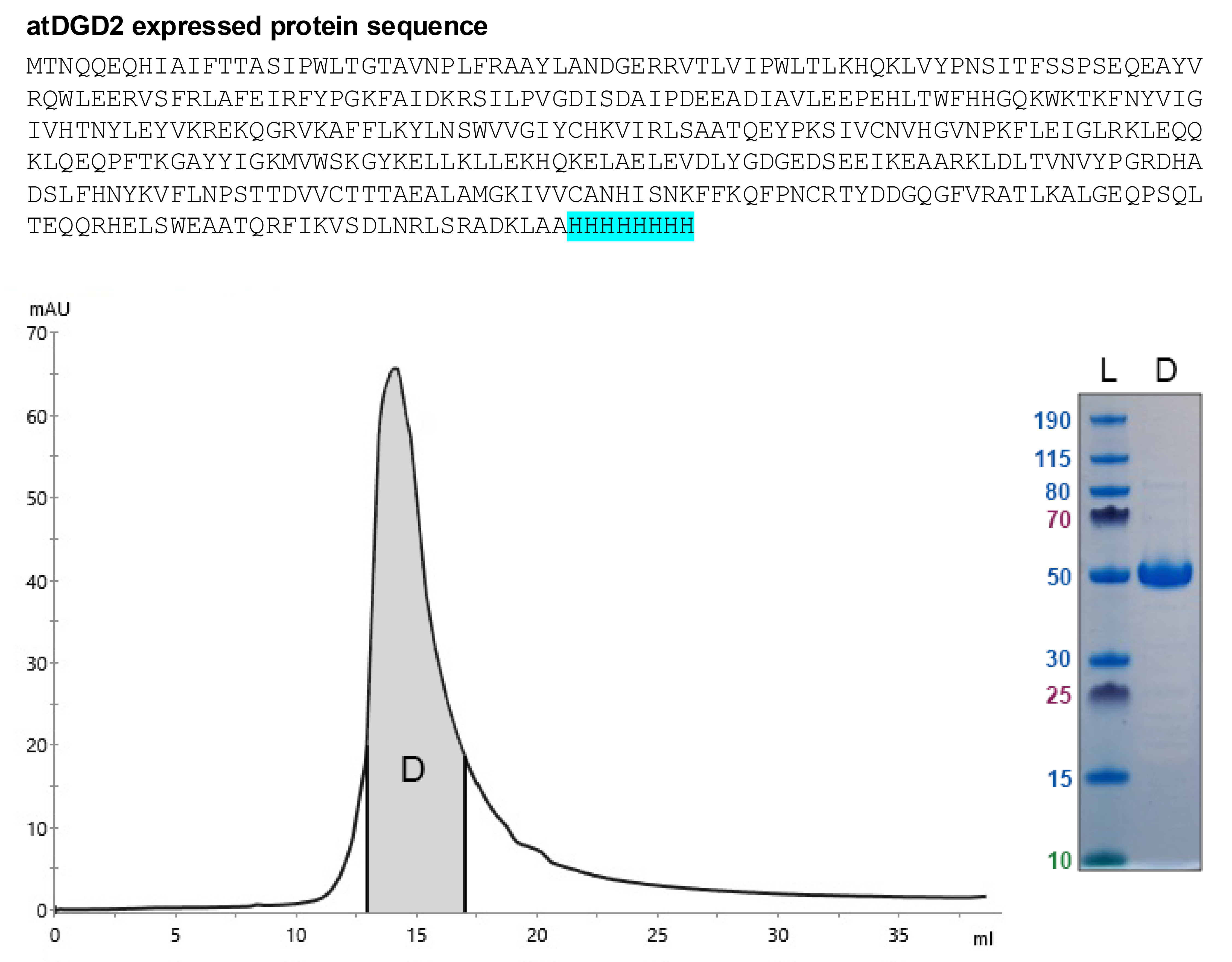
*

**Figure S9. Assessment of atDGD2(Δ401-473) purity following protein purification.** Upper panel: Protein sequence of expressed atDGD2(Δ401-473), which includes a non-cleavable C-terminal His-tag (highlighted in cyan). Lower panel: Chromatogram following Size-exclusion chromatography (SEC) using a Superdex200 10/300 increase column (Cytiva). 1 mg of protein was loaded onto the column. There was a single uniform peak (D), which eluted at the expected size of the atDGD2 monomer. The corresponding SDS-PAGE gel analysis for peak D is shown to the right of the chromatogram. L: PageRuler™ plus pre-stained protein ladder (Thermo Fisher Scientific).

**Table S1. Data collection and refinement statistics for atDGD2.**

| Data collection | | |
| --- | --- | --- |
| Beamline | PXII-SLS |  |
| Wavelength (Å) | 0.98 |  |
| PDB ID: | 8P6S |  |
| Space group | *P*4_1_2_1_2 |  |
| Cell dimensions |  |  |
| *a, b, c* (Å) | 44.0, 44.0, 368.5 |  |
| α, β, γ (°) | 90.0, 90.0, 90.0 |  |
| Resolution (Å) | 2.10 |  |
| Total reflections | 1308253 (32354) |  |
| Unique reflections | 22546 (1576) |  |
| Reflections used for R_free_ | 1154 (123) |  |
| R_merge_ | 0.146 (2.802) |  |
| R_pim_ | 0.025 (0.849) |  |
| CC(1/2) | 1.00 (0.488) |  |
| CC(work) | 0.943 (0.713) |  |
| CC(free) | 0.929 (0.619) |  |
| *I/σI* | 20.5 (1.4) |  |
| Completeness (%) | 99.1 (90.8) |  |
| Multiplicity | 58.0 (20.5) |  |
| Refinement | | |
| Resolution (Å) | 2.10 |  |
| No. reflections | 22244 (1154) |  |
| R_work_/R_free_ | 0.205/0.257 |  |
| No. atoms: |  |  |
| Protein | 2937 |  |
| Ligand/ion | 36 |  |
| Water | 60 |  |
| *B*-factors (Å^2^): |  |  |
| Wilson B-factor | 44.1 |  |
| Protein | 57.3 |  |
| Ligand/ion | 48.9 |  |
| Water | 52.3 |  |
| R.m.s. deviations: |  |  |
| Bond lengths (Å) | 0.014 |  |
| Bond angles (°) | 2.21 |  |
| Ramachandran plot (%): |  |  |
| Favored | 98.65 |  |
| Outliers | 1.35 |  |
| Clashscore | 7.0 |  |

**Table S2. Average RMS differences (Å) between the Ca atoms of atDGD2 (PDB ID: 8P6S, this work) and other glycosyltransferases (GTs).** *T. elongatus* Sucrose-phosphate synthase (teSPS), *M. smegmatis* phosphatidyl mannosyltransferase (PimA), *B. subtilis* glycosyltransferase BshA (bsBshA), *S. aureus* glycosyltransferase BshA (saBshA) and *A. thaliana* Monogalactosyldiacylglycerol (atMGD1). The residue ranges used for the calculations are shown in **Table S3**. Fructose-6-phosphate (F6P), Phosphotidylinositol (PI), Diacylglycerol (DAG). The donor sugar substrate of atDGD2 is UDP-galactose and the acceptor substrate is monogalactosyldiacylglycerol (MGDG).

| **Glycosyltransferase** | teSPS | PimA | bsBshA | saBshA | atMGD1 |
| --- | --- | --- | --- | --- | --- |
| PDB ID | 6kih | 4n9w | 5d00 | 6d9t | 4x1t |
| Donor substrate | UDP-glucose | GDP-mannose | UDP-*N*-acetylglucosamine | UDP-*N*-acetylglucosamine | UDP-galactose |
| Acceptor substrate | F6P | PI | L-malate | L-malate | DAG |
| **RMSD vs atDGD2** | teSPS | PimA | bsBshA | saBshA | atMGD1 |
| Whole monomer | 2.55 | 2.97 | 2.36 | 2.43 | 3.75 |
| N-terminal domain | 2.22 | 2.14 | 2.31 | 2.30 | 2.71 |
| C-terminal domain | 2.04 | 1.96 | 2.01 | 1.97 | 2.41 |

**Table S3. Residue range used for RMSD calculations in Table S2.**

| **Glycosyltransferase** | **PDB ID** | **Whole monomer** | **N-terminal domain** | **C-terminal domain** |
| --- | --- | --- | --- | --- |
| atDGD2 | 8p6s | 1-391 | 1-203 & 380-391 | 204-379 |
| teSPS | 6kih | 1-426 | 1-218 & 407-426 | 219-406 |
| PimA | 4n9w | 1-373 | 1-168 & 348-373 | 169-347 |
| bsBshA | 5d00 | 1-375 | 1-183 & 358-375 | 184-357 |
| saBshA | 6d9t | 1-373 | 1-171 & 355-373 | 172-354 |
| atMGD1 | 4x1t | 139-523 | 139-326 & 505-523 | 327-504 |

**Table S4. Important conserved residues between atDGD2 and other glycosyltransferases.**

| **atDGD2 residue** | **Role in atDGD2** | **teSPS** | **PimA** | **BsBshA** | **SaBshA** |
| --- | --- | --- | --- | --- | --- |
| Gly22  (N-term domain) | H-bonds with phosphate group of UDP | Gly50 | Gly16 | Gly16 | Gly13 |
| His151  (N-term domain) | H-bonds with galactose of UDP-Gal | His158 | His118 | His121 | His118 |
| Lys243  (C-term domain) | H-bonds with phosphate group of UDP | Lys254 | Lys202 | Lys212 | Lys209 |
| Glu321  (C-term domain) | H-bonds with ribose of UDP | Glu339 | Glu282 | Glu291 | Glu288 |

**References**

1. Baker, N. A., Sept, D., Joseph, S., Holst, M. J., and McCammon, J. A. (2001) Electrostatics of nanosystems: application to microtubules and the ribosome *Proc Natl Acad Sci U S A* **98**, 10037-10041

2. Pei, J., Kim, B.-H., and Grishin, N. V. (2008) PROMALS3D: a tool for multiple protein sequence and structure alignments *Nucleic Acids Research* **36**, 2295-2300
